# Emissivity Regulated Fabric: Achieving Self‐Adaptive Radiative Cooling and Dynamic Body Radiation Manipulation

**DOI:** 10.1002/smll.202504951

**Published:** 2025-08-05

**Authors:** Xin Hu, Yingbo Zhang, Wei Cai, Yang Ming, Rujun Yu, Daming Chen, Shuang Qiu, Cancheng Jiang, Chi‐Wai Kan, Jinlian Hu, Nuruzzaman Noor, Bin Fei

**Affiliations:** ^1^ Materials Synthesis and Processing Lab School of Fashion and Textiles The Hong Kong Polytechnic University Hung Hom Kowloon Hong Kong SAR 999077 P. R. China; ^2^ Research Centre for Resources Engineering Toward Carbon Neutrality The Hong Kong Polytechnic University Hung Hom Kowloon Hong Kong SAR 999077 P. R. China; ^3^ Department of Building Environment and Energy Engineering The Hong Kong Polytechnic University Hung Hom Kowloon Hong Kong SAR 999077 P. R. China; ^4^ Department of Material Science and Engineering City University of Hong Kong Kowloon Tong Kowloon Hong Kong SAR 999077 P. R. China; ^5^ Department of Biomedical Engineering City University of Hong Kong Kowloon Tong Kowloon Hong Kong SAR 999077 P. R. China

**Keywords:** dynamic radiative cooling, emissivity modulation, personal thermal management, phase transition

## Abstract

Effective manipulation of radiative cooling power is crucial for thermal management systems. However, the potential for radiative cooling regulation through emissivity modulation in textiles remains unexplored. As a proof‐of‐concept, a self‐adaptive radiative cooling fabric (SARCF) is presented, exhibiting high solar reflectance and variable infrared emissivity. The SARCF is created by depositing tungsten doped vanadium dioxide (W‐VO_2_) nanoparticles on low‐emissivity (low‐e) fabrics, followed by welding with nanoporous polyethylene (NanoPE). SARCF demonstrates significant solar reflectance (85.19%) and a promising emissivity contrast (Δɛ, 34.82%) for radiative cooling regulation, driven by the temperature‐induced phase transition of W‐VO_2_. Indoor and outdoor tests reveal that SARCF outperforms low‐emissivity fabrics and white cotton, providing better warming (3 °C higher than low‐emissivity fabrics) and cooling (4.67 °C lower than cotton) performance. The coated low‐e fabrics also demonstrated exceptional robustness—accelerated washing (10 cycles) retains >96% Δɛ, while abrasion test (2000 cycles) preserves 94.8% Δɛ, confirming mechanical integrity under operational stresses. In summary, this study introduces a novel fabric prototype that achieves temperature‐induced emissivity variation and high solar reflectance, marking a significant advancement in personal thermal management through radiative cooling modulation.

## Graphical abstract

Self‐adaptive radiative cooling textile dynamically switches radiative modes: Traps warmth in cold hours via low‐emissivity state, while boosting radiative cooling in heat hours through phase‐transition induced infrared emissivity modulation.

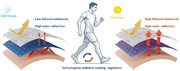

## 1 Introduction

Tremendous energy was consumed to provide thermal comfort for human in indoor environments annually via conventional heating, ventilation, and air conditioning systems, contributing to the global energy crisis and sustainability issues.^[^ ^1^, ^2^, ^3^, ^4^, ^5^, ^6^, ^7^, ^8^ ^]^ Passive personal thermal management concept, focusing on the manipulation of the human body local environment, was considered as a promising method to alleviate the massive energy consumption.^[^ ^9^, ^10^, ^11^ ^]^ The human body emits thermal radiation primarily in the midIR (Infared) wavelength range of 7–14  micrometer (µm), with peak intensity at around 9.5µm.^[^ ^12^ ^]^ Some reports have explored personal thermal management via manipulation of body IR radiation.^[^ ^13^, ^14^, ^15^, ^16^, ^17^ ^]^ For example, textiles for radiative cooling and radiative heating, have been developed to provide localized thermal control of the immediate environment around the human body.^[^ ^18^, ^19^ ^]^ Tong et al. proposed a theoretical design for an IR‐transparent, yet visibly opaque fabric made from synthetic polyethylene fibers.^[^ ^20^ ^]^ In addition to IR‐transmissive radiative cooling textiles, IR‐emissive radiative cooling textiles have also been developed by Zhu et al., proposing a nano‐processed silk with high emittance and solar reflection for radiative cooling purposes.^[^ ^10^ ^]^ As for radiative heating purpose, fabrics with strong IR reflection were fabricated for human body to remain warming. Hsu et al. developed a radiative heating fabric by embedding normal cotton with silver nanowires using a dip‐coating method.^[^ ^14^ ^]^

While these studies have demonstrated excellent performance in either single‐mode heating or cooling, they lack the ability to respond to changes in ambient environment. This may lead to thermal discomfort in some circumstances (radiative cooling in cold environments or radiative heating in hot environments). Therefore, dynamic reflectivity/emissivity regulation has attracted many researchers’ attention.^[^ ^21^, ^22^ ^]^ Few reports have explored dynamic IR radiation modulation via electricity or mechanical actuation.^[^ ^23^, ^24^, ^25^, ^26^, ^27^ ^]^ Zhang et al. realized the modulation of IR radiation as the relative humidity of underlying skin changed.^[^ ^28^ ^]^ They engineered triacetate‐cellulose bimorph fibers with a thin layer of carbon nanotubes to turn polymer fibers into electrically conductive. A maximum IR transmittance contrast of 35% can be achieved due to the resonant electromagnetic coupling effect induced by mechanical actuation, which was also triggered by sweating. Despite a few attempts at dynamic IR emissivity/reflectivity/transmissivity modulation for bidirectionally regulating of both heating and cooling, achieving temperature‐dependent IR emissivity manipulation in textiles without any energy input remains challenging.

In this paper, we developed a self‐adaptive radiative cooling fabric (SARCF) that can dynamically manipulate IR radiation. SARCF was prepared by depositing VO_2_ nanoparticles onto low‐e fabric substrates, supplemented by the lamination of an IR‐transparent nonporous polyethylene layer on top to provide the solar reflection and passageway for infrared. At low temperatures, SARCF exhibited a high solar reflection of 85.19% and a low IR emissivity of 39.38%, suppressing the radiative cooling effect. As the temperature increased, the IR emissivity shifted to 74.20%, enhancing the cooling effect. Such temperature‐dependent emissivity regulation can help humans to stay in thermal comfort against temperature fluctuations. Overall, this study introduces a facile approach for dynamic manipulation of radiative cooling power and body radiation via emissivity modulated fabrics, showing great potential in providing thermal comfort in various environments.

## 2 Results and Discussion

### 2.1 Design Concept and Fabrication of SARCF

**Figure 1:**
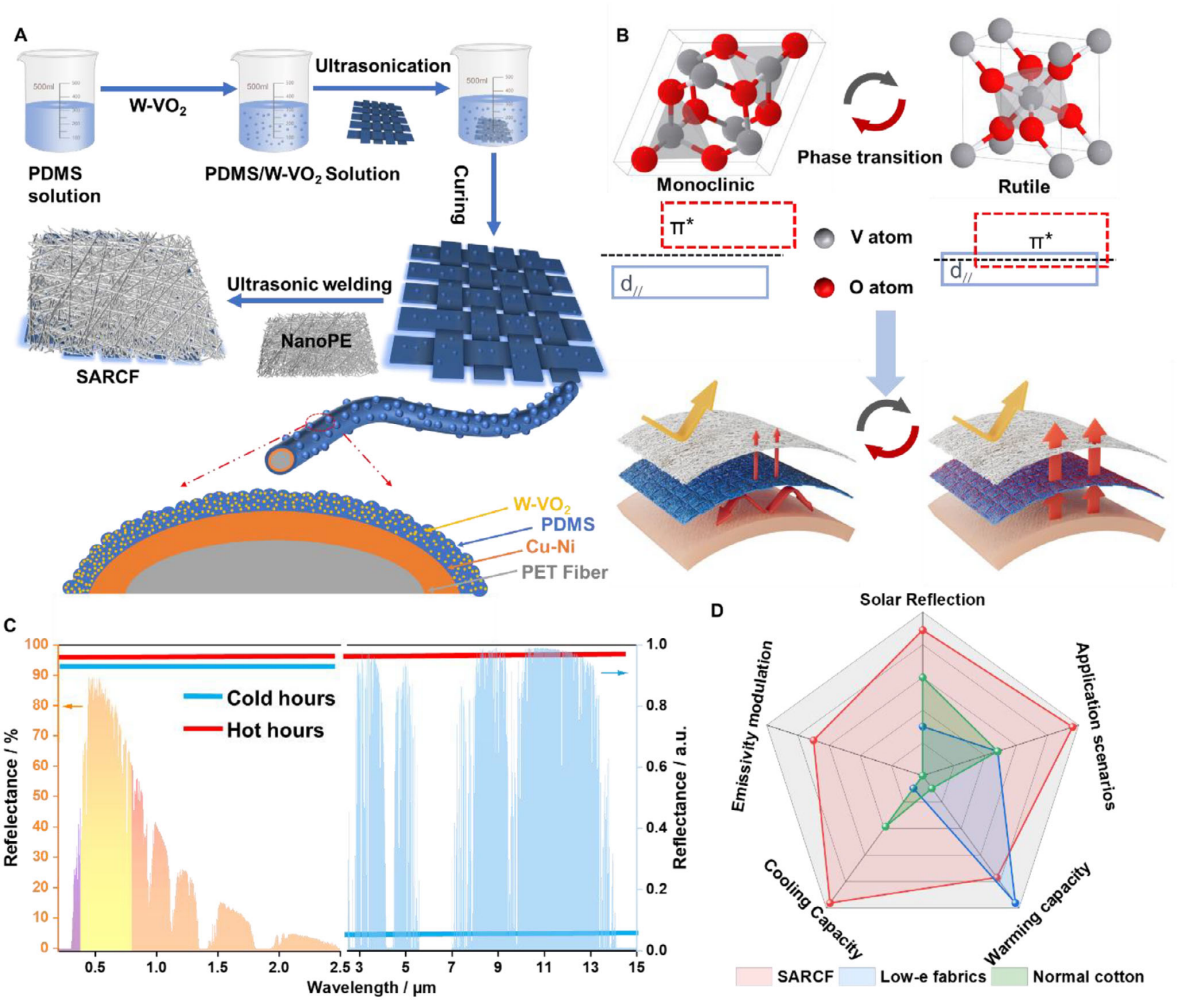
A) Schematic for the fabrication process of SARCF. B) Schematic for mechanism of the phase induced emissivity modulation process. C) The ideal spectrum of radiative cooling regulation fabrics. D) Radar chart for comparison in terms of solar reflection, application scenarios, cooling capacity, warming capacity, and emissivity modulation, among SARCF, low‐e fabrics, and normal cotton (Detailed assessment method can be found in Supplementary Note 1, Supporting Information).

The human body is a powerful infrared emitter, radiating energy at an intensity of 70–200 W m^−^^2^. Effectively managing this radiation is essential for maintaining thermal comfort. In cold environments, fabrics with low infrared emissivity (high reflectivity) are crucial as they reduce radiative heat loss, helping to keep the body warm. Conversely, in warmer settings, fabrics with high infrared emissivity (low reflectivity) aid in heat dissipation, keeping the body cool. Given the body's varying thermal needs across different environmental conditions, fabrics that can adjust their emissivity in response to ambient temperatures are highly advantageous. Ideally, a perfect self‐adaptive radiative cooling fabric should maintain high solar reflection irrespective of temperature changes, while offering temperature‐responsive infrared emissivity—low emissivity in cold conditions and high emissivity in hot conditions (**Figure** **1**C). To achieve this, we propose a straightforward strategy for developing fabrics with self‐adaptive radiative cooling regulation. SARCF consistently reflects solar radiation, while its emissivity adjusts to temperature changes, catering to the body's dynamic thermal needs in both cold and hot environments. The SARCF is fabricated by combining a lay of nanoporous polyethylene (NanoPE), which is solar‐reflective while infrared‐transparent layer (NanoPE), with an emissivity regulation layer (W‐VO_2_‐coated metallic PET fabrics), as illustrated in Figure 1A. The emissivity regulation layer (ERL) is constructed by depositing W‐VO_2_ nanoparticles onto metallic PET fabrics. These W‐VO_2_ nanoparticles undergo a metal‐insulator phase transition (Figure 1B), which alters their infrared properties, enabling self‐adaptive radiative cooling modulation. With its high solar reflectance and emissivity modulation capability, SARCF outperforms low‐emissivity fabrics and conventional cotton in providing thermal comfort under varying conditions (Figure 1D).

### 2.2 Temperature Induced Phase Transition of W‐VO_2_

**Figure 2:**
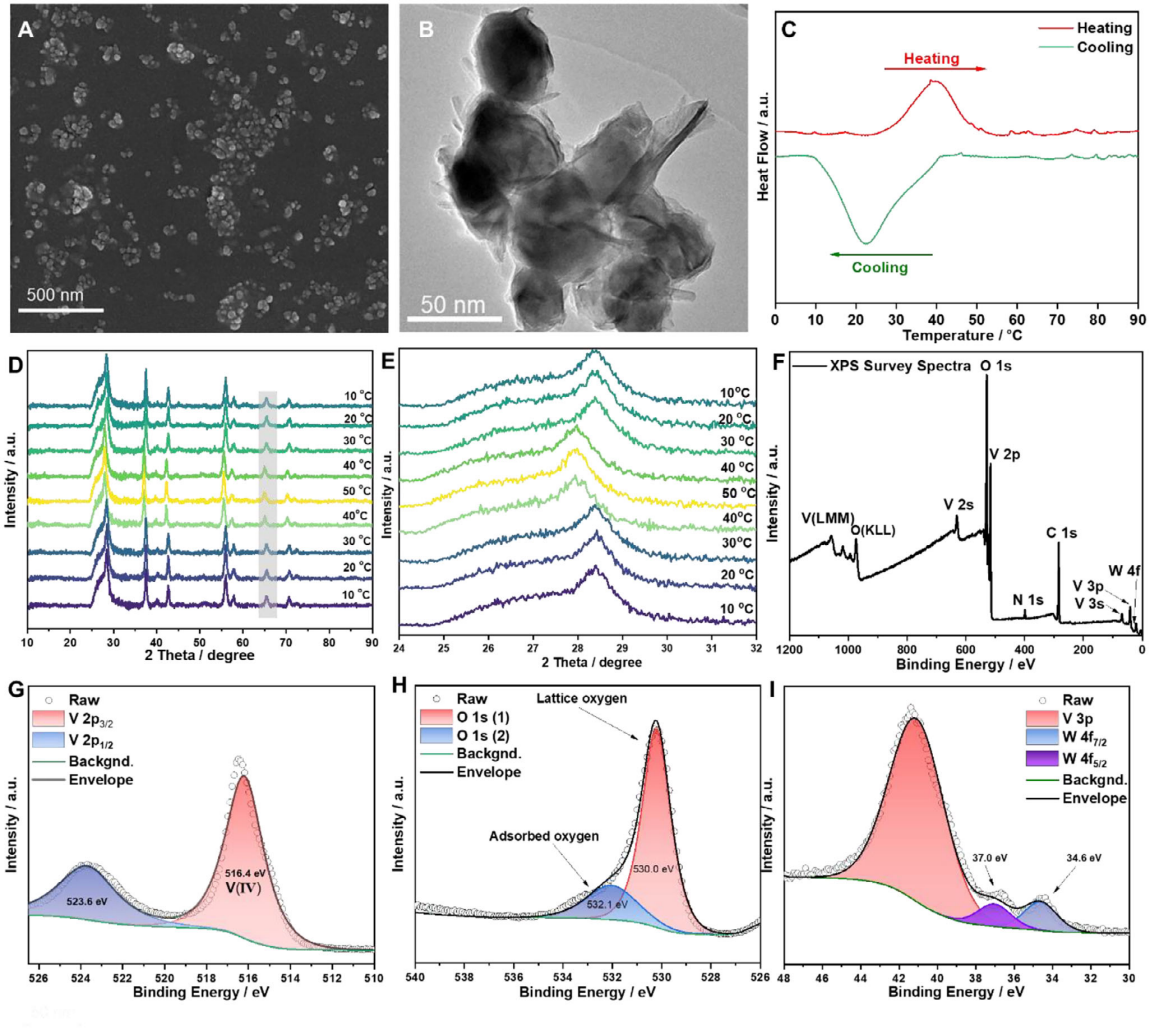
A)SEM and TEM B) images of W‐VO_2_ nanoparticles. C) DSC curve of W‐VO_2_ nanoparticles. D)Temperature‐dependent XRD patterns of W‐VO_2_ nanoparticles in 2 Theta of 10–90°. E) Enlarged temperature‐dependent XRD patterns of W‐VO_2_ nanoparticles from 24–32°. F) XPS survey W‐VO_2_ nanoparticles. G–I) High‐resolution scans for V2p, O1s, and W 4f.

Vanadium oxide (VO_2_) is a promising candidate for temperature responsive emissivity modulation due to its metal‐insulator phase transition behavior. More importantly, its transition temperature can be lowed to near room temperature (≈30 °C) via element doping (e.g., tungsten, fluorine, and magnesium), which is crucial to real word demonstrations.^[^ ^29^, ^30^, ^31^, ^32^, ^33^ ^]^ The tungsten doping concentration, nominally specified as 2% by the supplier (Supporting Information, Materials section), was quantitatively verified through independent characterization. Inductively coupled plasma optical emission spectroscopy (ICP‐OES) measurements yielded an average tungsten weight concentration of 3.65%, corresponding to an atomic ratio of 1.69% (Table S1, Supporting Information). As shown in SEM and TEM images (**Figure** **2**A,B), the size of the tungsten doped vanadium oxide (W‐VO_2_) nanoparticles is ≈30–50 nm. The endothermic peaks (39.2 °C) occur in the heating ramp in the DSC curve of W‐VO_2_, while exothermal (21.8 °C) peaks are observed during the cooling process (Figure 2C). The transition temperature(≈30.5 °C) was determined by averaging the temperature at exothermic and endothermic peaks, which is much lower than the undoped VO_2_ (≈68 °C). The near room temperature is considered to be more promising for the real word applications. Additionally, an apparent thermal hysteresis can be found in the DSC curves, which indicates the first order and reversible transition of W‐VO_2_.^[^ ^34^, ^35^, ^36^, ^37^ ^]^ The XRD patterns (Figure 2D) are well‐matched with the standard monoclinic phase (JCPDS No. 43–1051, VO_2_ (M)). The peak at around 28.4°, corresponding to (011) face, is the major feature of monoclinic phase.^[^ ^38^ ^]^ All other peaks can also be assigned to standard VO_2_ (M), no other additional phases were observed. The temperature‐dependent XRD (Figure 2D,E) patterns also confirmed the reversible phase transition (from the monoclinic VO_2_  (M) phase to the rutile VO_2_  (R) phase, as shown in Figure 1B). It can be observed that the strongest peak (001) at 28.4° was shifted to 27.9.^o[^ ^39^ ^]^ This is a clear indication of such structural phase transition, in which a VO_2_ (M) (011) transit into VO_2_ (R) (110). The detail effect of tungsten doping on the chemical composition of VO_2_ was investigated by XPS. The complementary XPS report on the element composition, after correction for adventitious carbon contamination, confirmed a tungsten doping level of 1.95 at% (Table S2, Supporting Information). These experimentally determined values (1.69‐1.95 at%) demonstrate excellent agreement with the nominal 2% doping concentration, also validating the consistency with DSC and XRD results. The All the spectra were calibrated based on the binding energy of 284.8 eV for C1s. As shown in Figure 2F, O1s, V3s, V3p, V2s, V2p, W4f, C1s, and N1s peaks were found in the XPS survey. Despite the binding energy of C1s was used as the reference for calibration, C1s and N1s peak are the contaminates introduced in the measurement process. V2p, O1s, and W4f peak were further deconvoluted in high‐resolution spectra for detail analysis, as shown in Figure 2(G–I). The fitting process was only conducted on the spin orbital splitting peaks of V2p, despite the scan range spans the O1s (overlapped with the V2p). Due to the O1s being not involved in the fitting process, the peak intensity ratio of V2p_3/2_ and V2p_1/2_ was manually set to 3:1. Consequently, the full width at half maximum (FWHM) of V2p_1/2_ was manually fixed to 1.5 times that for V2p_3/2_. As shown in Figure 2G, the two spin orbital splitting peaks of V2p (V2p_3/2_ and V2p_1/2_) can be observed, the binding energy of V2p_3/2_ (516.4 eV) was perfectly matched with V(IV). The splitting value observed (7.2 eV, 523.6–516.4 eV) was also different from that of V metal, which usually exhibited a splitting value of 7.6 eV. Therefore, the V(IV) can be confirmed. Two fitting peaks can be found in O1s spectra, one is adsorbed oxygen (532.1 eV, surface contamination), another is attributed to lattice oxygen (530.0 eV, V─O). In the case of W4f, the two orbital splitting peaks of W4f occur at 34.6 (W4f_7/2_) and 37 eV(W4f_5/2_), respectively, with a splitting value of 2.4 eV, which is inconsistent with the known splitting value of W metal (2.17 eV). This indicates that the existing form of W in the sample is W(VI).^[^ ^40^, ^41^, ^42^, ^43^ ^]^ Combining the DSC, DSC, XRD, and XPS results, we can rationally conclude that the transition temperature of monoclinic phase VO_2_ (M) was successfully lowered to 30.5 °C by tungsten doping.

### 2.3 Morphology of SARCF

**Figure 3:**
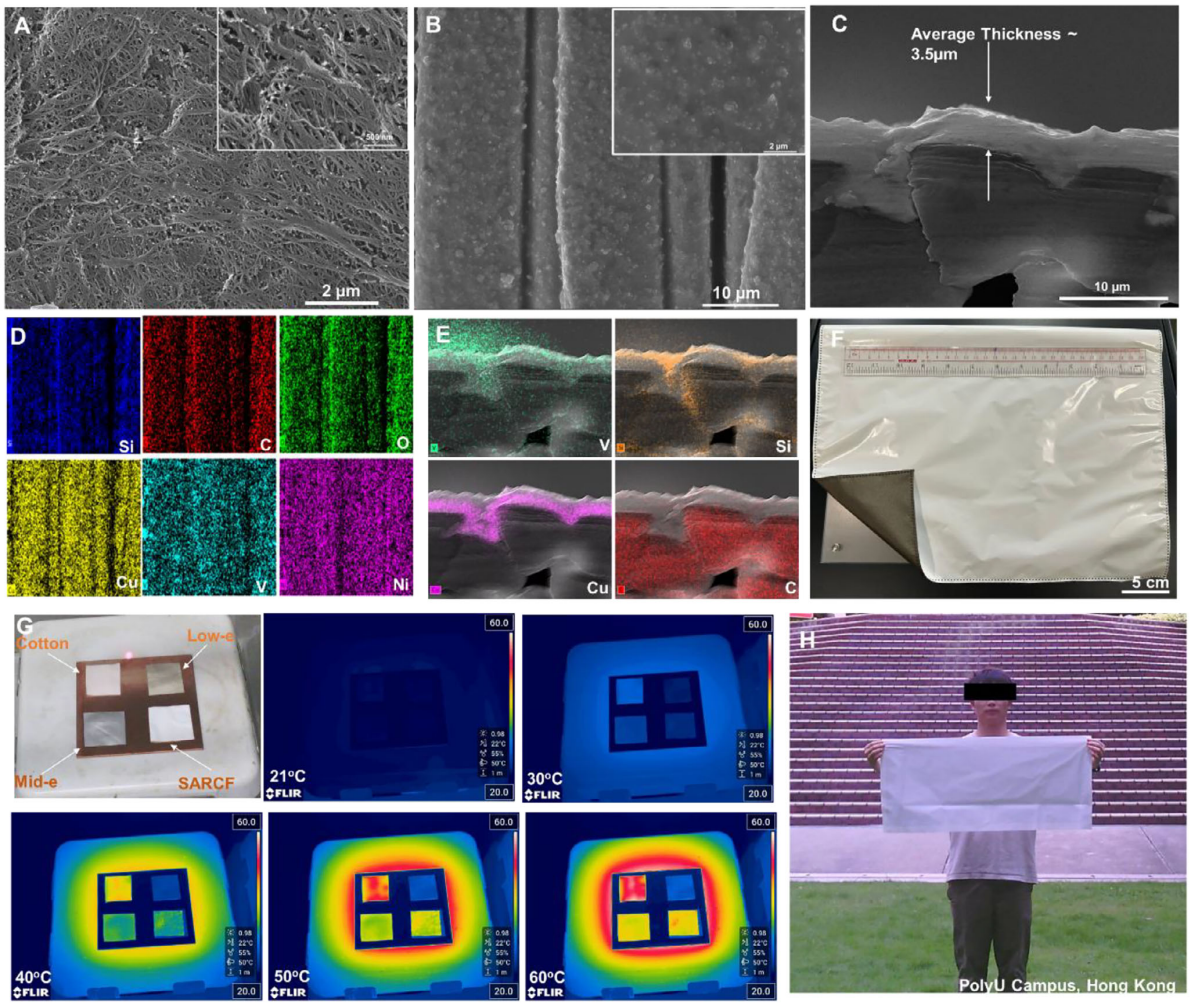
A,B) SEM images of the front side (NanoPE side) and back side (ERL)of the SARCF. C) SEM images of ERL from cross‐sectional view. D,E) EDS mapping of ERL. F) Digital image of SARCF. G) Digital image showing positions of cotton (top right), low‐e fabrics (top left), mid‐e fabrics (low left) and SARCF (low right), and IR images of these four samples at different temperatures (21, 30, 40, 50, and 60 °C). H) Digital image showing the scaling up potential of SARCF.

The SARCF mainly consists of two parts, the emissivity regulation layer in the bottom and the solar‐reflective but infrared‐transparent NanoPE shelter. The appearance of NanoPE is clearly white, which indicates considerable solar reflection. The integration of W‐VO_2_ nanoparticles onto low‐e fabric obviously changed its appearance, turning the color into light black (Figure 3F). The SEM images of NanoPE and ERL are shown in **Figure** **3**A,B. NanoPE is a widely adopted separator for lithium‐ion batteries, preventing the potential short circuit between anodes and cathodes. Therefore, NanoPE has interconnected porous structures in nano scale (50–1000 nm), resulting in more than 50% of the pore volume and strong Mie scattering in visible range. This will then lead to high solar reflection, while maintaining near‐perfect infrared transmittance, which is considered to pose little effect on the infrared properties of objective being covered by NanoPE. Therefore, the infrared emissivity of ERL underneath the NanoPE can be reserved, making the radiative cooling regulation possible. The ERLs were prepared by simple dip coating with the assistance of ultrasound. The SEM images of ERL3 were shown in Figure 3B, continuous coating consisting of W‐VO_2_ nanoparticles were observed. The particle density of W‐VO_2_ nanoparticles increased with the increase of W‐VO_2_ concentrations (Figure S3e, Supporting Information). However, the increase of the concentration also causes more agglomeration in the matrix(Figure S3D, Supporting Information). Th increased root mean square roughness (Rq, Figure S4, Supporting Information) also indicates the formation of particle aggregation. Si, C, O, Cu, Ni, and V were found in the EDS mapping images (Figure 3D), among which Cu and Ni resulting from the low‐e coating on PET fabrics, Si, C and O corresponding to the PDMS binder, V and O originating from W‐VO_2_ nanoparticles. The tungsten is not detected in the EDS mapping due to its low doping concentration. But its presence has been confirmed by the XPS spectra. The cross‐sectional image shows that the Cu‐Ni/W‐VO_2_ /PDMS coating is ≈3.5 µm (Figure 3C). Due to the strong adhesion of PDMS and limitation on sample preparation for SEM imaging, the boundary between low‐e coating and PDMS‐W‐VO_2_ is not clear. But the EDS mapping (Figure 3E) has clearly shown this boundary by the distribution of V and Cu elements. Then SARCF was then fabricated by assembling the ERLs and NanoPE via ultrasonic wielding (Figure 3F).

The IR properties of SARCF were first demonstrated via IR imaging. Normal cotton, low‐e fabrics, and commercial Middle‐emissivity (Mid‐e) fabrics (≈an emissivity of 0.56 within 7–14 µm, Figure S5, Supporting Information) were selected as a comparison. To ensure even heating, all samples were put on a cupper plate, which was sitting on a heater with temperature control. The scale bar was manually fixed to 20–60 degrees, while the reference emissivity was set to 0.98. The IR images at room temperature (≈21 °C), 30, 40, 50, and 60 °C were obtained respectively. As shown in Figure 3G, though four samples can be barely seen at room temperature, the normal cotton is still brighter than other samples. This indicates that the emissivity of cotton (≈0.87 within 7–14 µm, Figure S6, Supporting Information) is much higher than the other three samples. The low‐e fabrics and SARCF are clearly darker than cotton and Mid‐e fabric at 30 °C, indicating a lower emissivity. It should be noted that the SARCF is darker than Mid‐e fabric (≈an emissivity of 0.56), demonstrating the fact that the emissivity of SARCF is lower than 0.56. SARCF becomes a little bit brighter than Mid‐e fabric but is still darker than cotton at 40 °C, indicating that its emissivity has gradually surpassed the Mid‐e fabrics while remaining lower than cotton (≈0.87). It is clear that SARCF becomes brighter than Mid‐e fabrics at 50 and 60 °C, revealing its higher emissivity. The relative brightness of SARCF compared to Mid‐e fabrics experience a clear change, indicating the emissivity modulation capacity during the increases of the temperatures. The SARCF has the potential to be scaled up (Figure 3H), despite we only provide a proof of concept demonstration in this study. The scale‐up production demonstrated here was achieved by spaying coating.

### 2.4 Temperature‐Dependent Optical Properties of SARCF

**Figure 4:**
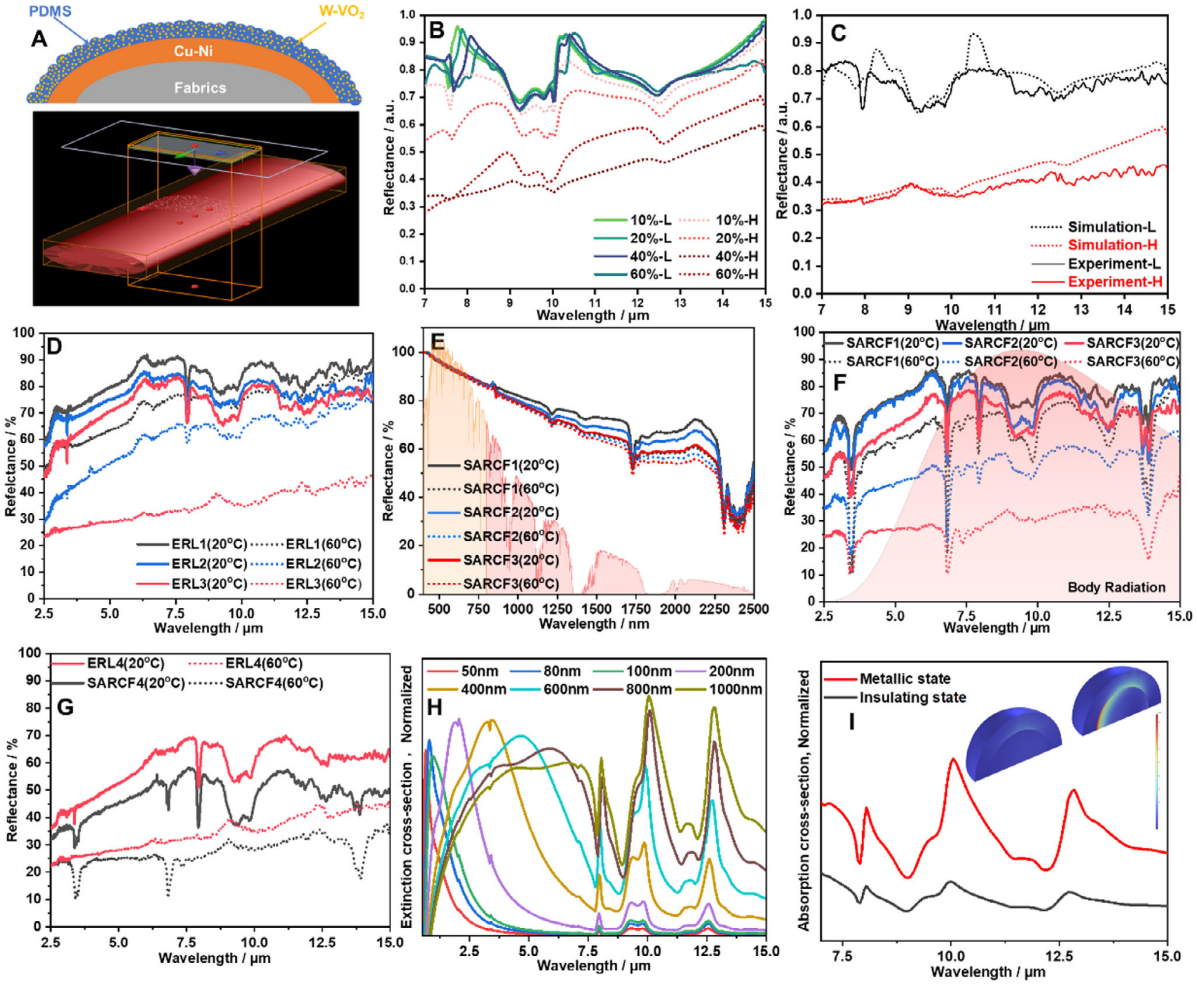
A) Schematic of the ERL model for FDTD simulation. B)simulated MIR reflectance at different fill rate of W‐VO_2_. C) Simulated(dash line) and experimental (solid line)reflectance of ERL3 at 20 °C (black) and 60 °C (red). D) The infrared reflectance of ERL1, ERL2, and ERL3 at 20 °C (solid lines) and 60 °C (dash lines). E) The solar reflectance of SARCF1, SARCF2, and SARCF3 at 20 °C (solid lines) and 60 °C (dash lines). F) Infrared reflectance of SARCF1, SARCF2, and SARCF3 at 20 °C (solid lines) and 60 °C (dash lines). G) Infrared reflectance of ELR4 at 20 °C (solid lines) and 60 °C (dash lines). H) Normalized Extinction cross section of W‐VO_2_ particles at different radius. I)Normalized absorption cross section of W‐VO_2_ (800 nm)in PDMS matrix(inserted figure, loss power density at insulating and metallic state).

As discussed before, Nanoporous PE was adopted to provide high solar reflection and thoroughfare for the emission from the ERL. As shown in Figure S7 (Supporting Information), NanoPE exhibited the typical absorption peaks of ‐CH_2_‐ at 3.44 (2920 cm^−1^), 6.8 (1460 cm^−1^), and 13.9 µm (720cm^−1^). Apart from these peaks, NanoPE showed high transmittance within other wavelengths. More importantly, these peaks are all outside of the atmospheric window. Overall, NanoPE exhibited high infrared transmittance of 96.87%, 90.56%, and 82.98%, at the number of layers of 1, 2, and 4, respectively. Such high infrared transmittance poses little impact on the infrared performance of materials below the NanoPE, thereby, facilitating the observation of emissivity modulation. As a result, the low‐e fabrics show an infrared transmittance of 94.14% when covered by a layer of NanoPE, while the original infrared reflectance is 99.23%. More impressively, low‐e fabrics can exhibit an infrared reflectance of 84.34%, despite covered by 4 layers of NanoPE (Figure S8, Supporting Information). The NanoPE also functions as the solar shelter for SARCF, providing considerable solar reflectance. As shown in Figure S9 (Supporting Information), four layers of NanoPE exhibit a solar reflectance of 83.56%, which is much higher than that of low‐e fabric (47.05%,Figure S9, Supporting Information). Interestingly, NanoPE shows high reflectance visible range, while low‐e fabrics show high reflectance near infrared range. The complementary reflection in visible and near infrared range may lead to high reflection in all wavelengths when integrating NanoPE and low‐e fabrics. The W‐VO_2_ nanoparticle coated low‐e fabrics were the emissivity regulating layer (ERL) for SARCF. Here, we assume that W‐VO_2_ nanoparticles were randomly distributed in the PDMS matrix and coated on the low‐e fabrics (**Figure** **4**A, top). The MIR (7–15 µm) properties of ERL (SARCF without Nano‐PE) were simulated at different filling ratio of W‐VO_2_ nanoparticles. The simulation model was shown in Figure 4A (bottom). The simulated MIR reflectance of ERL at insulating (‐L) and metallic (‐H) states were shown in Figure 4B. It is clear that the Δɛ is highly related to the filling ratio. The simulated Δɛ increased as the rise of filling ratio. The experimental results (ERL3) seemingly confirmed the simulated results (Figure 4C). The measured solar reflection of ERL experienced a considerable reduction, which is not favorable for radiative cooling applications, compared to the bare low‐e fabrics. As shown in Figure S10 (Supporting Information), the solar reflection gradually decreased as the increase of W‐VO_2_ content. In visible range, the reflectance of ERLs almost remain unchanged. Meanwhile, ERLs showed temperature‐adaptive reflectance in NIR band(1250–2500 nm), where the reflectance decreased when temperature rose up. However, the soar irradiance in 1250–2500 nm only accounts for ≈10.9% of the total solar energy, making the solar regulation efficiency limited. In the MIR range, the reflectance of ERLs decreased compared to the initial low‐e fabrics due to the introduction of PDMS binder. Importantly, both ERL1, ERL2, and ERL3 showed negative reflectance differences, i.e., positive Δɛ (Figure 4D). A broad absorption within the MIR was observed for ERLs at high temperature (Figure S11, Supporting Information), indicating higher emissivity regulation capacity. A maximum Δɛ of 36.24% (65.85% of infrared reflectance at 20 °C and 29.61% at 60 °C) can be achieved for ERL3. It should be noted that the high infrared reflectance of low‐e fabric substrate is crucial to achieve negative reflectance difference (i.e., positive Δɛ) that meets the concept of radiative cooling regulation. Even though the same fabrication process of ERL3 was applied to bare PET fabrics, negative reflectance difference cannot be obtained (Figure S12, Supporting Information).

After the preparation of ERLs, four layers of NanoPE ultrasonically wielded onto the ERLs to construct SARCF. Similar to ERLs, the solar reflection of SARCF decreased with the increase of VO_2_ content (Figure 4E). Meanwhile, the solar reflection of SARCF1 was 86.80% at 20 °C, while this value decreased to 84.78% when W‐VO_2_ transitions to rutile state at 60 °C (Figure 4E). SARCF2 and SARCF3 also experienced similar reduction as the rise of temperatures. The limited solar modulation s is due to two fact that 1) the self‐adaptive reflectance of ERLs mainly occurred in 1250–2500 nm band and 2) NanoPE film is highly solar reflective in this waveband. These will then cause any changes in solar reflectance on ERLs will be invisible for the detector of the spectrometer. Though the solar modulation of SARCF is limited, the decent modulation on infrared properties can be reserved due to the high MIR transmittance of NanoPE. The infrared reflectance of SARCF1, SARCF2, and SARCF3 at different 20 and 60 °C were shown in Figure 4F. All the SARCFs exhibited different levels of infrared reflectance modulation. Specifically, SARCF1 shows an infrared reflectance of 71.21% at 20 °C, while its infrared reflectance falls to 55.29% at 60 °C, corresponding to an Δɛ of 15.92%. The Δɛ increases to 26.51% (an infrared reflectance of 68.50% at 20 °C and 41.99% at 60 °C) for SARCF2. The maximum Δɛ was achieved by SARCF3, which exhibits an infrared reflectance of 60.62% at 20 °C and 25.80% at 60 °C, corresponding to an Δɛ of 34.82%. When focus was put on atmospheric window (8‐13 µm) range, the Δɛ can be more impressive, reaching up to 38.46%. Further increase in the content of W‐VO_2_ cannot result in high Δɛ. The infrared properties of ERL4 and SARCF4 were shown in Figure 4G. Detailly, ERL4 shows an Δɛ of 21.93%, which is lower than that of ERL (36.24%). Similarly, the Δɛ of SARCF4 was also lower than that of SARCF3, falling from 34.82% to 18.01%. The bottom face of SARCF3 can be considered as ERL3, showing high infrared reflectance (65.85%) at low temperatures and avoiding body heat loss. While it exhibits low infrared reflectance (29.61%) at high temperatures, promoting body heat dissipation. The upper face of SARCF3 were characterized by high solar reflection as well as temperature‐dependent infrared optical properties. The solar reflection reaches 85.19% at 20 °C and then decreases to 84.24% at 60 °C, which makes the daytime radiative cooling possible. More importantly, its infrared emissivity is 39.38% at 20 °C (i.e., infrared reflectance of 60.62%), which is beneficial to maintain the body temperature at cold environment. However, its infrared emissivity then rises to 74.20% at 60 °C (i.e., infrared reflectance of 25.80%), promoting the body heat dissipation. The optical properties of SARCF meet the requirements of wearables in both cold and hot environments, and the transition is induced passively by the ambient temperature without energy input.

### 2.5 Explanation for the Modulation

Despite the decent emissivity modulation capacity demonstrated in this study, the detailed mechanism behind this still remains unclear. First of all, there is no doubt that modulation is induced by the temperature. However, the detailed physics of the temperature‐induced phase transition still remains unclear. We will not explore the detailed physics about the phase transition, but a brief explanation for the experimental results obtained in the study. From previous research, the most widely accepted one is a multilayer model, i.e., Fabry‐Perot resonator, which consists of a bottom reflective layer (usually metal), a dielectric layer and VO_2_ layer. Specifically, the infrared property of the F‐P structure was determined by the bottom reflective layer when the temperature is below the phase transition temperature as the VO_2_ layer is transparent to infrared at this phase. While the temperature is above the phase transition temperature, the bottom reflective layer, and the dielectric layer and the top metallic VO_2_ layer can be considered as a typical F‐P cavity, which leads to strong absorption enhancement at certain wavelengths due to interference. Tang's^[^ ^44^ ^]^ work has clearly demonstrated constructive interference and destructive interference at certain wavelengths in a VO_2_ based F‐P resonator theoretically and experimentally. It should be noted that VO_2_ was considered a pure thin film their demonstration. However, randomly distributed VO_2_ particles in PDMS matrix might be the most appropriate model to describe the real situation in this study. The broadening of the absorption peaks in the metallic W‐VO_2_/PDMS composite film at elevated temperatures may arise from the size‐dependent localized surface plasmon resonance (LSPR) characteristics of the embedded metallic W‐VO_2_ particles. As evidenced by the extinction cross‐section analysis (Figure 4H), the LSPR peak of metallic VO_2_ particles exhibits a pronounced redshift with increasing particle size, transitioning from 600 nm (near‐infrared, NIR) for 50 nm‐radius particles to 7.5 µm (mid‐infrared, MIR) for 1000 nm‐radius particles. This polydispersity in particle dimensions within the composite system leads to spectral overlap of resonant absorption bands across distinct size populations, consequently resulting in a broadened absorption profile spanning both NIR and MIR regions under thermal activation conditions. We can also observe the increased absorption and scattering within 7–15 µm when the temperature was elevated (Figure 4I; Figure S13, Supporting Information). The amplified scattering events induce multiple light‐particle interactions, effectively elongating the optical path length and promoting energy dissipation through cumulative optical losses. Critically, the PDMS matrix exhibits intrinsic strong absorption across this spectral range, ensuring efficient capture of the scattered radiation. Concurrently, the metallic VO_2_ particles themselves demonstrate intrinsic absorption in the MIR region due to their characteristic electronic and lattice vibrational transitions. The combined effects of (1) PDMS‐mediated absorption of scattered photons and (2) direct MIR absorption by metallic VO_2_ collectively drive a pronounced broadband absorption enhancement in the 7–15 µm range. The amplified scattering and absorption mechanism and red shift of LSPR peak under thermal activation led to the considerable Δɛ in this study.

### 2.6 Thermoregulatory Performance of SARC in Different Environments

**Figure 5:**
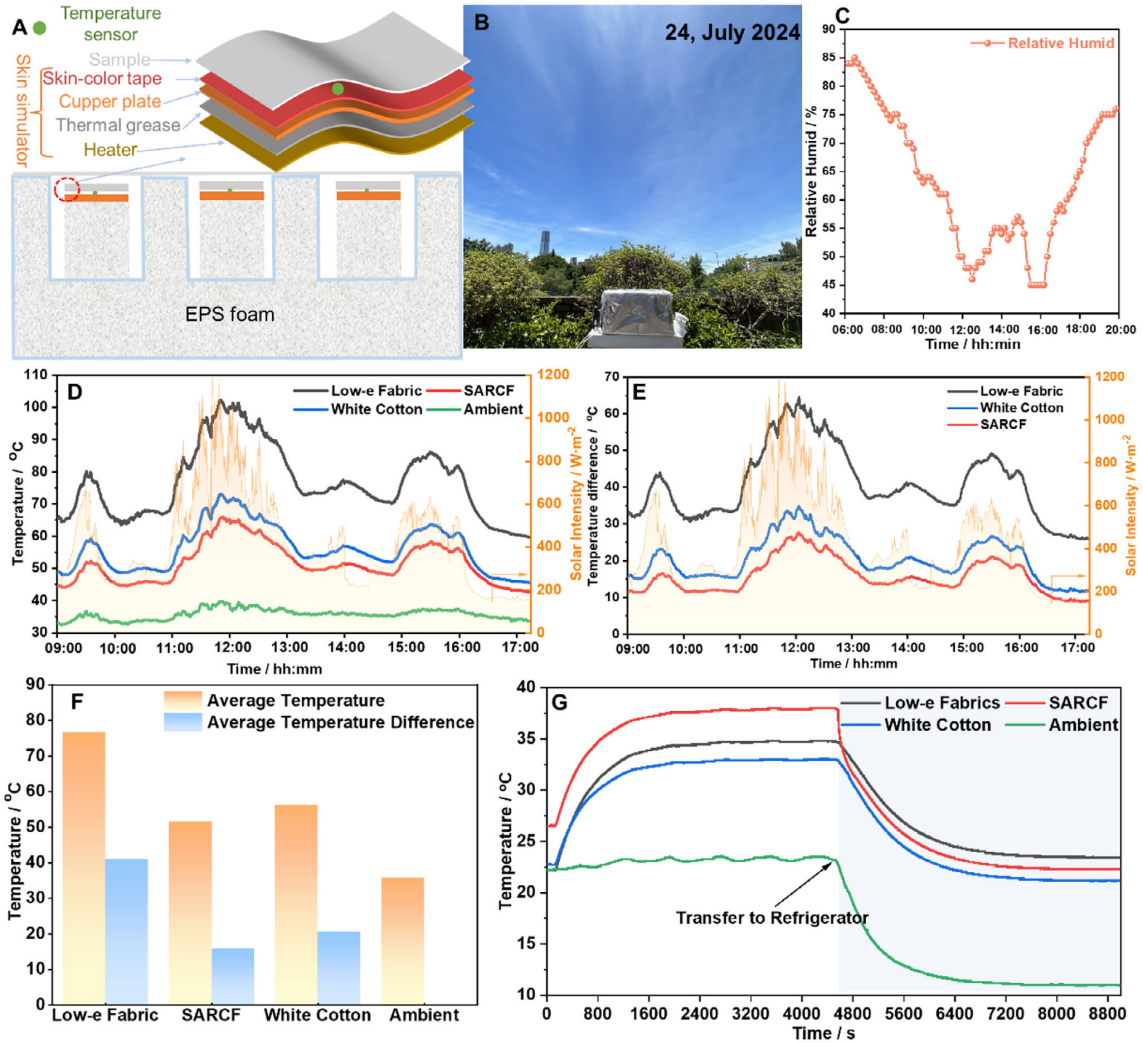
A) Schematic of setups for the outdoor test indoor test and the multilayer structure of the skin simulator. B) Sky images when conducting the outdoor test. The test was conducted on July 24, 2024. C) Relative humidity when conducting the outdoor test. D) The real‐time surface temperature of the skin simulator during the outdoor test. E) Temperature difference compared to ambient temperature during the outdoor tests. F)Average temperature and average temperature difference during the test. G) Temperature changes during the indoor test.

The optical properties have indicated that SARCF have great thermal management potential in both cold and hot environments. Here, the thermal performance of SARCF was evaluated in two typical environments, which includes outdoor and indoor, with self‐made devices (**Figure** **5**A,B). Two typical textiles (commercial white cotton and low‐e fabrics) were chosen for comparison. The optical properties of commercial white cotton can be found in Figures S14,S15 (Supporting Information). Specifically, low‐e fabrics show a solar reflectance of 47.05% (Figure S10, Supporting Information), and a near unit infrared reflectance (99.23%, Figure S8, Supporting Information). The commercial white cotton exhibits a solar reflectance of 67.62% (Figure S14, Supporting Information), a solar transmission of 27.08% (Figure S14, Supporting Information), and an infrared emissivity of 77% within 2.5–15 µm (Figure S15, Supporting Information). SARCF3 was chosen for the thermal measurement due to the maximum achievement of Δɛ. All these textile samples were integrated into similar measuring setups, which consisted of a skin simulator, a PT100 temperature sensor (adhered to the upper surface of skin simulator to monitor the temperature of skin simulator in real‐time manner), and insulating foam. The skin simulator was carefully designed, a cupper plate was used as the heat diffuser, and the space between the cupper plate and the heater was filled with thermal grease to ensure effective heat transfer. To simulate the optical properties of real human skin, a 3 m skin‐color tape was pasted on the cupper plate. The final skin simulator shows a solar reflection of 47.58% and an infrared emittance of 87.84% (Figure S1, Supporting Information), which are similar to those of real skin. To simulate the metabolic heat production rate of human skin, a constant input power of 150 W m^−2^ was applied to the heater. The electrical resistance of heater adopted is strictly limited to 17.5 ± 0.1Ω to ensure that temperature differences was caused by different optical properties rather than input power. The PT 100 temperature sensors were also subjected to calibration before use, ensuring the accuracy of temperature data.

As shown in Figure 5B, the outdoor test was conducted in July 24, 2024, a cloudy day in Hong Kong. The moisture around the experimental setups was recorded during the test (Figure 5C). A peak solar irradiance of 1199 W m^−2^ and the highest ambient temperature of 39.5 °C were recorded at 11:45 and 11:50 AM, respectively. The average ambient temperature during the monitoring period was 35.74 °C. As shown in Figure 5D,E, the SARCF covered skin simulator has the lowest surface temperature among there textiles. The surface temperature of the skin simulator is highly related to the solar irradiance, which indicates the solar reflectance of the fabrics dominates the temperature of the localized thermal microenvironment. The low‐e fabrics show the lowest solar reflectance (47.05%), therefore, the temperature covered by the low‐e fabrics was up to more than 100 °C with a temperature peak of 102.3 °C. Such high temperature is caused by the high solar absorption (a solar absorption of 52.95%) and high infrared reflectance, which result in significantly solar heating effect and preventing infrared radiation loss from the simulator, respectively. This performance is not acceptable for wearable applications, despite the sweating cooling system of the human body is not considered. For the case of white cotton, the surface temperature of the skin temperature is much lower than that of low‐e fabrics due to its much lower solar absorption (5.3%, a solar reflectance of 67.62% and a solar transmission of 27.08%). It is also deserved to note that solar transmission will also lead to photothermal effect on the surface of the skin simulator, thereby, causing the rise of surface temperatures. Despite the lower surface temperatures of the skin simulator covered by white cotton compared to low‐e fabrics, the peak temperature was still up to 73.2 °C. Therefore, white cotton may not be the best option in outdoor daytime. The surface temperature of the skin simulator covered by SARCF was further decreased compared wo white cotton throughout the test, this is mainly due to high solar reflection (84.24%) under sunshine. Due to the ambient temperature is always higher than 30 °C, therefore, SARCF was also considered as high‐emissivity state, which may promote the body heat loss by infrared radiation. The peak of the surface temperature covered by the SARCF was still up to 65.3 °C, which was much lower than those of low‐e fabrics (102.3 °C) and white cotton(73.2 °C.). Despite all the average surface temperature of the skin simulator being obviously higher than ambient temperature (35.74 °C), the average surface temperature of the skin simulator covered by SARCF (51.60 °C) was significantly lower than those covered by low‐e fabrics (76.67 °C) and white cotton (56.27  °C) during the test (Figure 5F). This indicates that SARCF might be better choice to maintain thermal comfort in outdoor daytime compared to low‐e fabrics and commercial white cotton.

Similar setups were adopted to further evaluate the thermal performance of SARCF in cold hours. The skin simulators were again covered by SARCF, low‐e fabrics, and white cotton. The surface temperature of the skin simulator was first stabilized in room temperature (22 °C). The whole setup was then transferred into a refrigerator with an ambient temperature of (5 °C) to simulate the cold environment. As shown in the Figure 5G, the surface temperatures of the skin simulator covered by SARCF, low‐e fabrics, and white cotton were stabilized at 37.8, 34.8, and 33 °C, respectively, in room environment. Due to the exclusion of solar irradiance for indoor environment, the surface temperature of the skin simulator was mainly determined by the infrared properties and the thermal conductivity. The surface temperature of the skin simulator covered by white cotton stabilized at 33.1 °C under room temperature (22 °C), while that covered by low‐e fabric stabilized at 34.8 °C, 1.7 °C higher than cotton. This is mainly due to the higher infrared reflectance of low‐e fabrics, which prevents the radiation transfer the low‐e fabrics and ambient. While commercial white cotton can be considered as an emissive type of radiative cooling textile, thereby promoting the heat loss from the skin simulator. The thermal conductivity is also a crucial factor. The low‐e fabric can be considered as an excellent conductor for heat, as a result, the heat generated by the skin simulator can be easily transferred to the upper surface. Then such heat can be dissipated into ambient through thermal convection. While the thermal conductivity of white cotton is lower than low‐e fabrics, which will then result in better warming effect compared to low‐e fabrics from a perspective of thermal conduction and thermal convection. Therefore, the warming effect of low‐e fabrics via infrared radiation is offset by the cooling resulting from thermal conduction and thermal convection. Similarly, the warming effect of SARCF was also strengthened by its lower thermal conductivity, which resulted from the air gaps between ERLs and NanoPE, and also the nanopores in NanoPE. Consequently, the stabilized surface temperature of the skin simulator covered by SARCF surpass that covered by low‐e fabrics under room temperature (22.0 °C), reaching 37.8 °C. When the experiment setups were transferred into refrigerator, the ambient temperature drastically fell to 5.0 °C. However, the detected ambient temperature fell to 11.0 °C and remained stable. This might be caused by the thermal insulation foam, which acts as a thermal barrier between the refrigerator (5.0 °C) and inside thermal insulation foam box. The temperature inside the insulation box (11.0 °C) is the result of a thermal equilibrium between the insulation box and the ambient temperature of the refrigerator space (5.0 °C). After transferring into refrigerator, the surface temperature of all skin simulators gradually decreased with the decrease of the ambient temperature inside the box, reaching 23.4 °C (low‐e fabrics), 22.3 °C (SARCF), and 21.2 °C (Cotton), respectively. This is seemingly conflicting with the conclusion obtained from final stabilized temperature under room temperature, in which it demonstrates that the warming effect of SARCF was strengthened via thermal conduction and convection. The possible reason for the fact that the final stabilized temperature of the skin simulator covered by SARCF is lower than that covered by low‐e fabrics is the room space allows strong thermal convection while the refrigerator space does not. The offsetting on the warming effect (obtained infrared radiation reflection) of low‐e fabrics via thermal convection is decreased, infrared radiation dominated the heat transfer process again. Therefore, the skin simulator covered by low‐e fabrics exhibited a higher final stabilized surface temperature compared to SARCF due to its high infrared reflectance. However, SARCF still outperforms the white cotton under this condition.

### 2.7 Wearability and Durability

**Figure 6:**
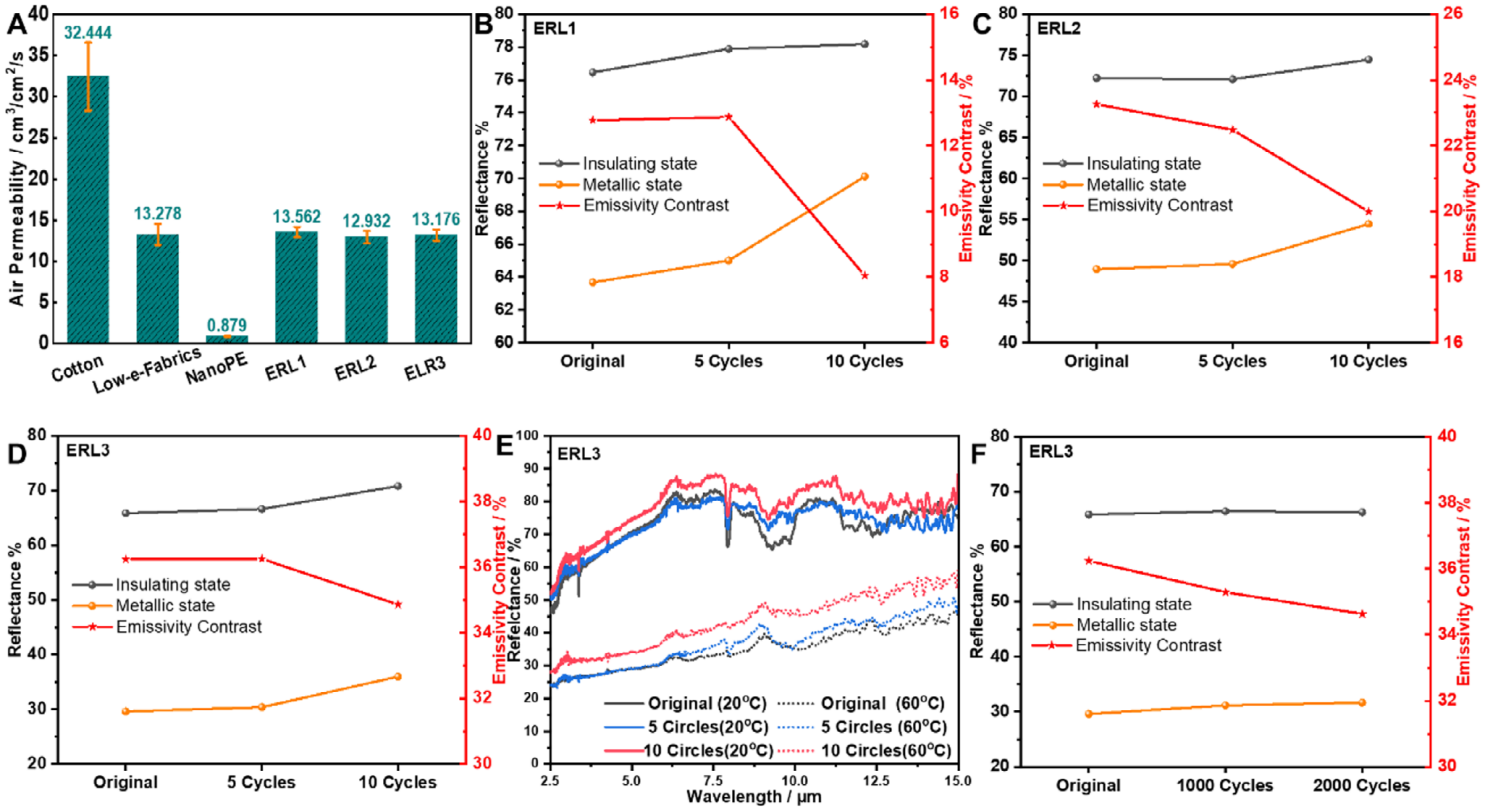
A) Air permeability of cotton, low‐e fabric, ERLs and NanoPE. B–D) The average infrared reflectance of ERLs at insulating and metallic states, and corresponding emissivity contrast after 0, 5, and 10 cycles of washing. E) The infrared reflectance spectrum of ERL3 at insulating and metallic states, and corresponding emissivity contrast after 0, 5, and 10 cycles of washing. F) The average infrared reflectance of ERL3 at insulating and metallic states, and corresponding emissivity contrast after 0, 1000, and 2000 cycles of washing.

The wearability and durability are the important concerns for practical applications. Here, the air permeability, washing and abrasion durability were evaluated. The air permeability of the fabrics using an SDL ATLAS Air Permeability Tester under ambient conditions (22 °C, 60–70% RH). The results (**Figure** **6**A) confirm that the inherent construction of the low‐emissivity fabric (low‐e fabric, 13.278 cm^3^ cm^−2^ s^−1^) and ERL samples results in lower air permeability compared to cotton (low‐e/ERL vs cotton: 13.278/13.562 vs 32.444 cm^3^ cm^−^^2^ s^−1^). Crucially, the application of the PDMS/VO_2_ coating showed no significant reduction in air permeability compared to the uncoated low‐e fabric. Figure S4 (Supporting Information) further visually confirms that the PDMS coating preserves the pore structure within the fabric substrate. This indicates the coating's limited impact on the fabric's existing breathability characteristics. While the measured air permeability of the ERL fabrics is lower than cotton, we maintain that this level is acceptable for wearable applications in indoor environments, where direct solar irradiance is absent and thermal management relies primarily on regulating IR emissions. The preserved porosity and measured permeability suggest adequate comfort under these conditions. However, the adoption of NanoPE shield pose limitation on the breathability: its inherently low air permeability (0.879 cm^3^ cm^−^^2^ s^−1^) is insufficient for conventional textile comfort standards. Its inclusion was necessary solely to address the critical requirement of solar reflectance for effective radiative cooling under outdoor conditions. Its IR transparency enables the underlying ERL's variable emissivity to function. Despite this, the integration of NanoPE for outdoor conditions is still valuable.

An accelerated washing test was conducted to reveal the washing durability of ERLs. Figures 3B–E reveals a gradual increase in infrared reflectance for both material phases alongside a concomitant decrease in Δɛ over the washing cycles. This trend indicates a degree of mechanical abrasion or partial removal of the VO_2_/PDMS functional coating during agitation, potentially leading to localized exposure of the underlying low‐emissivity fabric substrate. Crucially, however, the degradation rate was relatively low, and the core functionality was well‐preserved after 10 cycles. ERL1 retained a Δɛ of 8.05% (compared to its initial value of ≈12.77%), ERL2 maintained a Δɛ of 19.99% (initial ≈23.26%), and notably, ERL3 exhibited exceptional durability, retaining a Δɛ of 34.86% – representing 96% of its original high performance (≈36.24%). This minimal reduction, particularly for the highest‐performing ERL3 configuration, demonstrates that the structural integrity of the PDMS matrix and its adhesion to both the VO_2_ particles and the fabric substrate remain largely intact under repeated mechanical stress and aqueous exposure. The robust retention of emissivity modulation, especially for ERL3, confirms that the VO_2_/PDMS coating possesses substantial washing durability suitable for practical implementation in wearable thermal management textiles. In addition to the washing durability, the abrasion durability of ERL was also assessed by Martindale Abrasion and Pilling Tester (Figure 6E; Figure S16, Supporting Information). Following 2000 cycles, ERL3 exhibited excellent mechanical resilience. Infrared reflectance increased marginally from 65.85% to 66.29% in the insulating state and from 29.61% to 31.65% in the metallic state. This resulted in a slight reduction of the emissivity contrast from 36.24% to 34.36%, retaining 94.8% of its original Δɛ value. Crucially, the post‐abrasion emissivity contrast remains exceptionally high (>34%) and fully capable of enabling effective dynamic thermal regulation. In summary. the minimal performance degradation in terms of optical properties under sustained mechanical stress and washing demonstrates robust interfacial adhesion of the VO_2_/PDMS composite to the textile substrate, affirming its potential for practical wearable applications.

## 3 Conclusion

In this study, we pioneered the concept of self‐adaptive radiative cooling fabrics. As a proof of concept, emissivity regulation layers (ERLs) were fabricated by depositing W‐doped VO_2_ nanoparticles onto inherently low‐emissivity fabrics. A significant infrared emissivity contrast of up to 36.24%, driven by the insulator‐to‐metal phase transition of W‐VO_2_, enables effective radiative cooling regulation in indoor environments. After integrating these ERLs with a nanoporous polyethylene (NanoPE), the resulting fabric achieved a high solar reflectance of 85.19% alongside a substantial emissivity contrast of 34.82%, thus demonstrating its capability for dynamic radiative cooling manipulation under sunlight. Durability testing reveals exceptional robustness–‐accelerated washing (10 cycles) retains >96% emissivity contrast (Δɛ) in ERL3, while abrasion test (2000 cycles) preserves 94.8% Δɛ, confirming mechanical integrity under operational stresses. Overall, this work successfully demonstrates the viability of utilizing VO_2_ phase transition for self‐adaptive radiative cooling regulation in textiles, providing a promising pathway toward more intelligent personal thermal management solutions. Nevertheless, vanadium biosafety and wearing comfort remain important considerations for practical applications.

## Conflict of Interest

The authors declare no conflict of interest.

## Supporting information



Supporting Information

## Data Availability

The data that support the findings of this study are available from the corresponding author upon reasonable request.
