# Supplementary material for: Emissivity Regulated Fabric: Achieving Self‐Adaptive Radiative Cooling and Dynamic Body Radiation Manipulation
Source: Small. 2025 Aug 5;21(38):e04951. doi: 10.1002/smll.202504951 (PMC12462578; doi:10.1002/smll.202504951)
Supplement: Supplementary file 1 — Supporting Information [file SMLL-21-e04951-s001.docx]

**Supporting Information**

**Emissivity Regulated Fabric: Achieving Self-Adaptive Radiative Cooling and Dynamic Body Radiation Manipulation**

Xin Hu^1,2^, Yingbo Zhang^3^, Wei Cai^1,2^, Yang Ming^1,2^, Rujun Yu^1,2^, Daming Chen^1,2^, Shuang Qiu^1,2^, Cancheng, Jiang^4^, Chi-Wai Kan^1^ , Jinlian Hu^5^, Nuruzzaman Noor^1,2^, Bin Fei ^1,2^*

1 Materials Synthesis and Processing Lab, School of Fashion and Textiles, The Hong Kong Polytechnic University, Hung Hom, Kowloon, Hong Kong SAR, 999077, China.

2 Research Centre for Resources Engineering towards Carbon Neutrality, The Hong Kong Polytechnic University, Hung Hom, Kowloon, Hong Kong SAR, 999077, China.

3 Department of Building Environment and Energy Engineering, The Hong Kong Polytechnic University, Hung Hom, Kowloon, Hong Kong SAR, 999077, China.

4 Department of Material Science and Engineering, City University of Hong Kong, Kowloon Tong, Kowloon, Hong Kong, SAR, 999077, China

5 Department of Biomedical Engineering, City University of Hong Kong, Kowloon Tong, Kowloon, Hong Kong, SAR, 999077, China

**Methods**

Materials

Tungsten doped vanadium dioxide (W-VO_2_) was purchased from Hangzhou Jikang New Materials Co., Ltd. The particle size of the W-VO_2_ nanoparticles (NP) was approximately 30-50 nm. The tungsten doping concentration is ~2%, which was informed by the supplier. Low-e fabrics with Cu-Ni coating were purchased from Guangzhou Jiujing electronic materials Co., Ltd. The thickness of the low-e fabrics is 0.06mm with a surface resistance of <1 Ω. The bare polyester fabrics were obtained from corroding the Cu-Ni via diluted nitric acid (Sigma-Aldrich, 60%). Nanoporous polyethylene (Nano-PE, 25 µm) was bought from SK Innovation. Polydimethylsiloxane (PDMS, SYLGARD 184) was bought from Dow Co., Ltd.

Fabrication of self-adaptive radiative cooling regulation fabric

The self-adaptive radiative cooling regulation fabric (SARCF) was prepared by decorating low-e fabrics with randomly distributed W-VO₂ NP and then stitching this coated fabric together with nanoPE. The VO₂ NP were coated onto low-e fabrics via dip coating with sonication. Specifically, various amounts of W-VO₂ were added to an isopropanol solution (50 ml) containing 1 g of PDMS with sonication. The mixture was sonicated for 5 hours to ensure complete and even dispersion of the W-VO₂ NP. Subsequently, 0.1 g of curing agent was added to the mixture, which was then sonicated for an additional 30 minutes. The Low-e fabrics were immersed in the mixed solution for 15 minutes under ultrasound. The W-VO₂ coated fabrics was obtained after drying in a vacuum oven at 80°C for 24 hours. Finally, the SARCF was obtained by assembling the W-VO₂ coated fabrics and NanoPE together via ultrasonic wielding.

Thermal measurement

The thermal regulating capacity of the prepared SARCF was assessed using the experimental setup depicted in Figure. 5A. To simulate human skin, a layered structure comprising a Kapton heater, thermally conductive silicone grease, a copper plate, and 3M tape were employed. The infrared (IR) properties of this skin simulator closely mimic those of real human skin, as shown in Figure. S1. The Kapton heater, powered by a direct current source, delivered a heat flux of approximately 150 W/m²[1], equivalent to the metabolic heat generation of the human body. The silicone grease and the 1.5 mm thick copper plate functioned as a thermal connector and heat diffuser, respectively, ensuring uniform temperature distribution across the surface. To minimize external thermal influences during testing, aluminum foil and EPS foam were integrated into the device. Temperature data was continuously recorded every second using a M2100 Series (Samcq) Temperature Acquisition Remote IO Module, which was connected to a laptop and equipped with PT100 resistance thermometers to monitor the temperature. Thermal evaluations were conducted in both hot and cold environments. For the hot environment, tests were performed at noon in Hong Kong, where the ambient temperature exceeded 35°C. In contrast, the cold environment tests took place inside a refrigerator, maintaining an ambient temperature of 5°C.

Infrared (IR) imaging

The IR images were collected using a FLIR E95 (working wavelength ~7-14 µm, emissivity reference ~0.98), where a copper background with a ε_7-14_ of 0.1was employed. Four samples, white cotton, low-emissivity (low-E) fabric, heat-shielding fabric, and SARCF were placed on the copper plate, which sat on a heater with temperature control. Note that environmental radiation, especially human body radiation, has a significant impact on the imaging of low-E materials due to its high IR reflectance. Therefore, the whole setup was put in an acrylic that insulated against the external radiation. The heater ramp rate was held at every temperature point for 5 min to avoid possible error caused by the different thermal conductivity of each sample, and to stabilize readings. At the same temperature, the colour of the IR image indicates the emissivity in the working wavelength of the IR camera; darker colours suggest lower emissivity and vice versa[2].

Characterization

Temperature dependent XRD measurements were conducted on *Rigaku SmartLab* 9kW-Advance X-ray diffractometer equipped with an Anton PAAR TTK 600 temperature control system. XRD patterns within a heating-cooling (10-50-10^o^C) cycle were obtained to show the phase change of W-VO_2_. For each temperature point, signals were collected in the range of 10-90° with step size of 0.02° and scan rate of 10°/min using a HyPix-3000 Hybrid Pixel Array Detector. To ensure an accurate and stable sample temperature and associated structure stabilization, the XRD patterns were collected five minutes after the instrument temperature reached the set temperature point. The transition temperature of W-VO_2_ was measured by differential scanning calorimetry (DSC) on PerkinElmer DSC 8000. The DSC curve was treated by subtracting baseline and smoothing in Origin Pro 2022. The sample (<5 mg) was sealed in an aluminum pan and placed in the furnace. The sample and the reference pan were heated/cooled under nitrogen atmosphere (50 mL·min^−1^) with the heating/cooling rate of 20°C·min^−1^ from -10 to 100°C. The transition temperature was determined by averaging the corresponding temperatures of the two DSC peaks. Scanning electron microscope (SEM) images were obtained from TESCAN VEGA. All samples were coated with Au via a gold sputtering system before observation. The topography of the ERLs was obtained by a laser scanning microscope (KEYENCE VK-X200), and images were taken over a 1400*1000 µm area of the profile. Transmission electron microscopy (TEM) and high-resolution TEM (HRTEM) were conducted on JEOL 2100F, Japan (operating voltage = 200 kV). X-ray photoelectron spectroscopy analysis (XPS) was conducted by using ThermoFisher-nexsa with a monochromatic Al Kα X-ray source. The XPS survey spectrum as collected in the range of 0-1350 eV under the pass energy of 100 eV with the energy step size of 1 eV. The high-resolution spectra of V 2p, W 3p, and O 1s were obtained at a step size of 0.1 eV. For all XPS collection, the spot size of the x-ray is 400 µm. All data was collected absent any sputtering and all analysis was done on the Advantage software (Version 5.952) using C 1s (284.8 eV) as reference. The quantitative analysis of tungsten was obtained by the Agilent 7700(MS)

inductively coupled plasm-optical emission spectrometer (ICP-OES). A radio frequency power of 1.20kW was adopted. The reflectance (R(λ)) and transmittance (T(λ)) across 2.5-15 μm were measured by Fourier Transform Infrared (FT-IR Spectrometer, *Spectrum 100, PerkinElmer*) with a diffuse gold integrating sphere. The scanning range is 650 cm^-1^ to 4000 cm^-1^ at the resolution of 4 cm^-1^, and a total of 16 scans were collected. The reflectance and transmittance at high temperatures were obtained using a custom-made silicone rubber heating plate. The emissivity (ε(λ)) was calculated according to Kirchhoff’s law:

ε(λ) = A(λ) = 1-R(λ)-T(λ) - - - - - - - - (1)

where A(λ) and R(λ) are the absorbance spectrum and reflectance spectrum of the surface, respectively, while T(λ) is the transmission spectrum. The ε(λ) of SARCF was directly determined by the following formula:

ε(λ) = A(λ) = 1-R(λ) - - - - - - - - (2)

due to the negligible transmission T(λ) over the spectra of interest.

The integrated emissivity was calculated by averaging the absorbance spectrum ε(λ) within the corresponding wavelength. The maximum emissivity contrast (∆ε_max_) was determined by the following formula:

$$\Delta\varepsilon={\varepsilon_{high temperature}}-\varepsilon_{low temperature}$$

The temperature of the sample was controlled by a custom-made heating unit with temperature controller. The in-situ reflectance R(λ) across 200-2500 nm were obtained from SHIMADZU UV-3600 Plus with a 150 mm integrating sphere at 20^o^C and 60^o^C.

$$Rsol=\int_{200}^{2500} \varphi_{sol}(\lambda)\cdot R(\lambda)d\lambda/\int_{200}^{2500} \varphi_{sol}(\lambda)d\lambda$$

where the φ_sol_ (λ) is the solar irradiance spectrum distribution for AM 1.5 (corresponding to the sun standing 37º above the horizon with 1.5 atmosphere thickness and the presence of a solar zenith angle of 48.2^o^). Other spectra over the solar range without temperature control were collected from LAMBDA 1050+ UV/Vis/NIR spectrophotometer using a calibrated diffuse reflectance standard (Spectralon® reflectance standard, Labsphere) as a reference. It should be noted that the solar reflectivity reported in this work is a relative value to the reference. When the reflectivity of a sample exceeds that of the reflectance standard, the measured spectral reflectivity value is greater than one. To not overestimate the reflectivity of the sample, any measured spectral reflectivity that exceeded one was truncated to 1 during data processing. When nanoPE was involved, it can be noted that the reflectivity over UV range is greater than one, therefore, the reflectivity was truncated to 1 in this range.

The air permeability test was conducted according to ASTM D737, the test head is 5 cm^2^ and the pressure drop was set to 125.0 pa. For each sample, the air permeability was obtained by averaging five reads based on different sites of each sample. The washing durability evaluated by simulated washing test. The simulated washing protocol involved agitating the ERLs in a solution containing 5 g/L detergent for sequential 1-hour cycles, with each cycle representing one equivalent domestic wash. Following cycles 5, and 10, we quantitatively assessed the coating integrity by measuring infrared reflectance and calculating the emissivity contrast (Δε) between the insulating and metallic states. The abrasion durability was assessed by a Martindale Abrasion & Pilling Tester, employing wool fabric as the abradant. Optical properties, specifically infrared reflectance spectra and derived emissivity contrast (Δε) between insulating and metallic states, were evaluated at baseline and after 1,000 and 2,000 cycles to quantify coating integrity

Optical simulation

The optical characteristics of the fabricated structure (Figure 4A) within the 7-15 µm spectral range were numerically investigated through finite-difference time-domain (FDTD) simulations implemented in Lumerical FDTD Solutions. The computational model comprises a copper-coated polyethylene terephthalate (PET) fiber substrate overlaid with a vanadium dioxide (VO_2_)-polydimethylsiloxane (PDMS) composite layer, where VO_2_ nanoparticles were stochastically dispersed within the PDMS matrix. A parametric study was conducted to examine the influence of VO_2_ filler concentration, with volume fractions varying systematically at 10%, 20%, 40%, and 60%. Notably, the simulation protocol permitted particle aggregation and overlap effects to approximate real-world composite morphologies. The numerical domain was constructed by applying periodic boundary conditions along the x- and y-axes to simulate an infinite array configuration, while perfectly matched layer (PML) boundary conditions were implemented in the z-direction to mitigate artificial reflections. A normally incident plane wave excitation was imposed at the upper boundary of the computational space. Material optical constants were derived from established references: VO_2_[3], PET[4], Cu[5], and PDMS[4], with all constituent materials assumed to exhibit ideal surface morphology and chemical purity in accordance with common simulation practice. Surface roughness effects and impurity-related scattering mechanisms were intentionally excluded from the model to isolate the fundamental structure-property relationships.

The scattering characteristics of VO₂ particles embedded within a polydimethylsiloxane (PDMS) matrix were numerically investigated via finite element simulations (COMSOL Multiphysics 6.3). A parametric study was conducted by varying the radius r of the spherical VO₂ inclusions. To ensure computational efficiency while maintaining accuracy, the PDMS domain was bounded by a Perfectly Matched Layer (PML) to truncate the simulation space. This PML configuration effectively suppresses artificial boundary reflections without perturbing the electromagnetic solution within the region of interest, thereby ensuring consistency with results obtained from an unbounded domain. The total scattered energy was quantified through flux integration across a designated monitoring surface (Surface S). The incident wavefront was modeled as a transverse electromagnetic (TEM) wave propagating along the positive x-axis, with electric field polarization aligned parallel to the z-axis. Symmetry boundary conditions were strategically applied to reduce computational complexity: Perfect Magnetic Conductor (PMC) conditions were enforced on the x-z symmetry plane, while Perfect Electric Conductor (PEC) conditions governed the x-y symmetry plane. These boundary treatments enabled efficient simulation of the periodic scattering system. As schematically illustrated in Figure. S3, the geometric model incorporates randomly distributed VO₂ particles approximated as equivalent spherical scatterers within the PDMS host medium, establishing a representative volume element for analyzing effective scattering responses.

​Supplementary Text for Figure 1D:

The radar chart in Figure 1D provides a qualitative comparison of SARCF, low-emissivity (low-e) fabric, and cotton across five performance parameters. Quantitative data standardization was applied as follows: Solar reflection values were normalized relative to SARCF (assigned 100%), with other fabrics scaled proportionally to their measured reflectivity values. Warming and cooling capacities were benchmarked against field-test temperature differentials, assigning SARCF and low-e fabric maximum scores of 100 for cooling and warming capabilities respectively. Emissivity modulation utilized absolute values, plotting SARCF at its measured Δε of 34.82% against a 0-50% axis range, while low-e fabric and cotton scored zero due to their static infrared properties. Application scenario versatility employed categorical classification, designating SARCF for dual-mode operation (cooling/warming), low-e fabric exclusively for warming, and cotton solely for cooling applications.


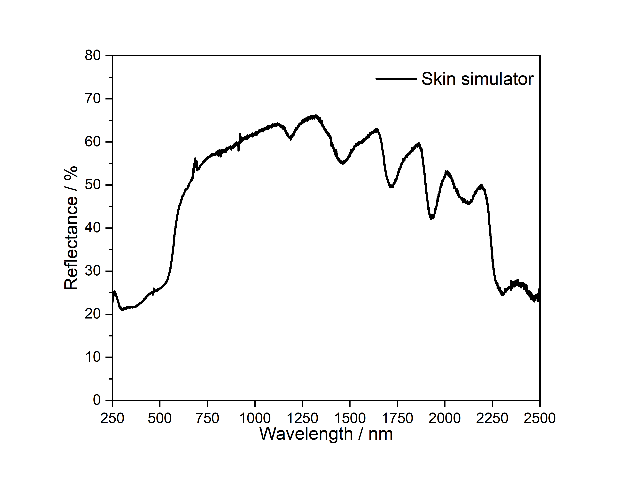

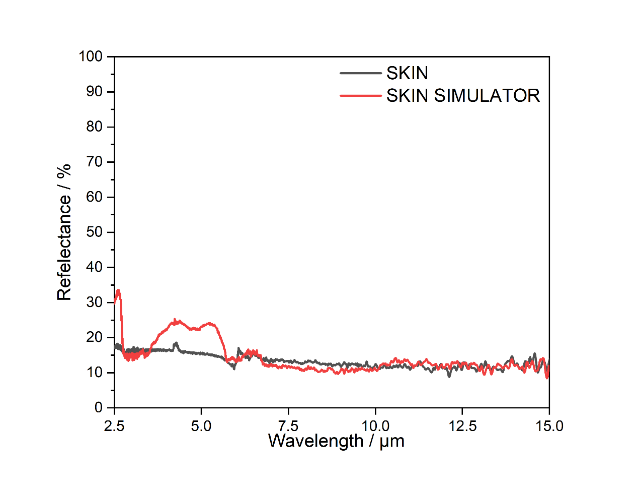


Figure. S1. Infrared reflectance and solar of real skin and skin simulator.

**
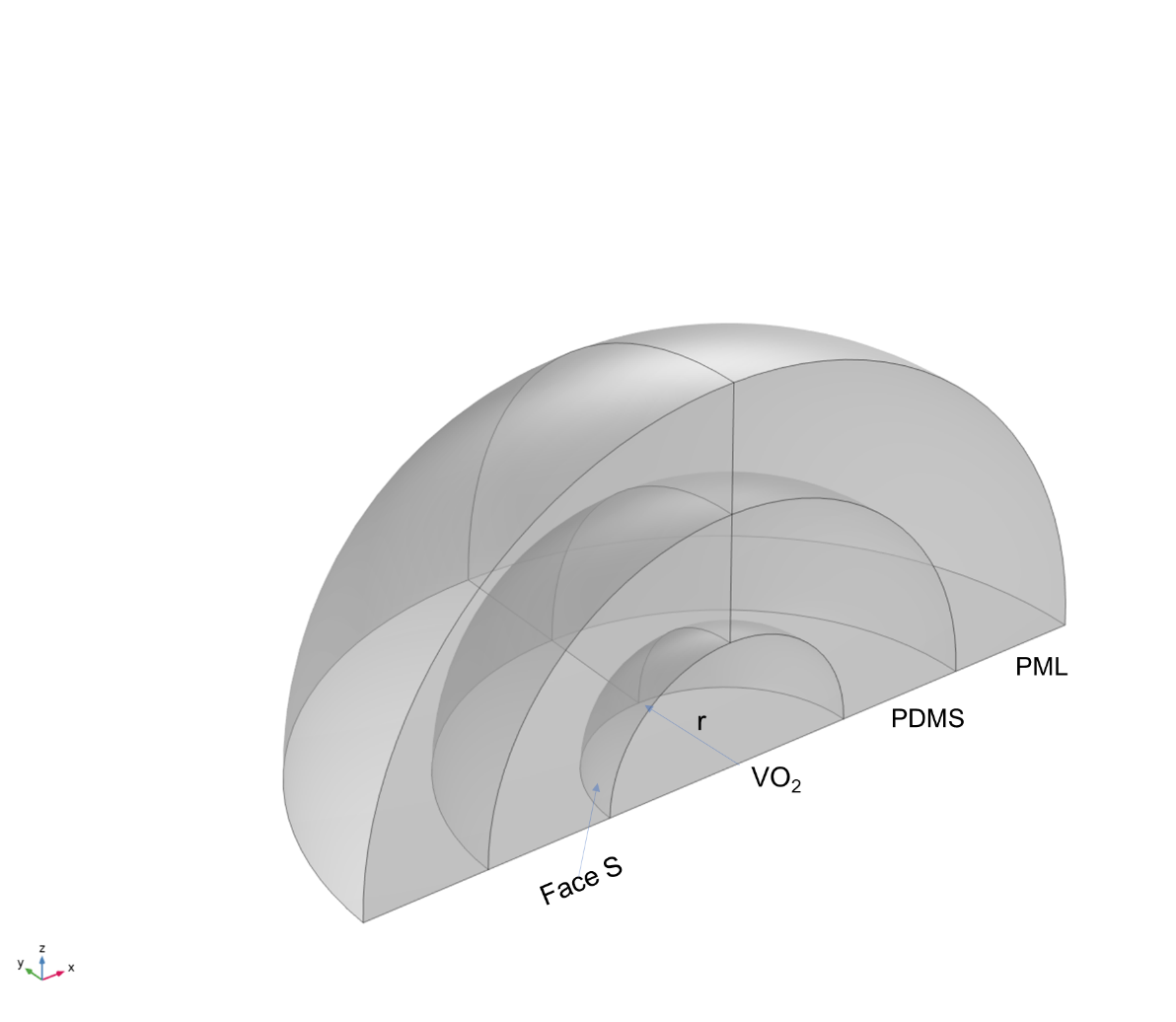
**

Figure. S2. Geometric diagram of equivalent sphere VO_2_ particle spherical particle scattering model.


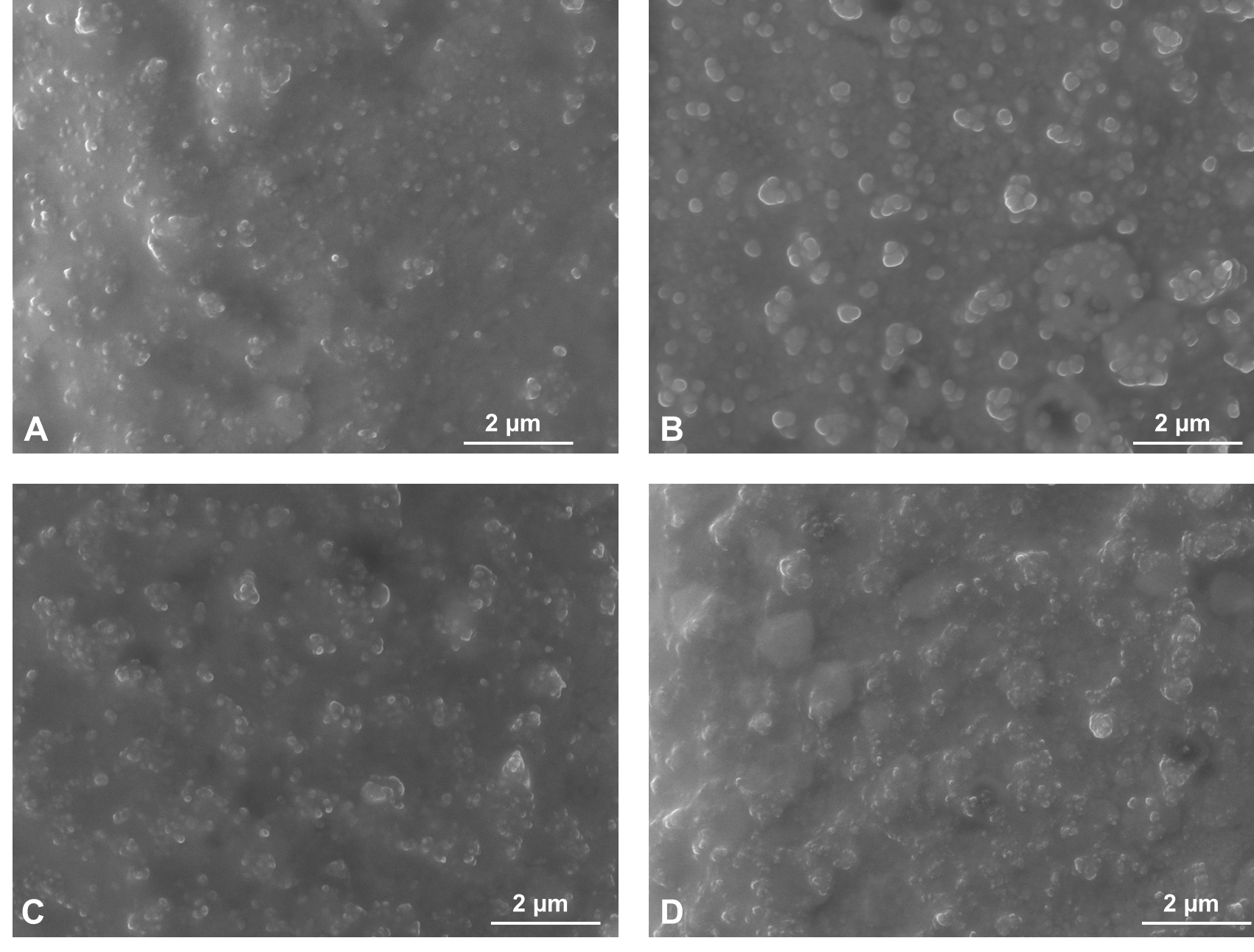


Figure. S3. SEM image of ERL1 (A), ERL2 (B), ERL3 (C) and ERL4 (D).


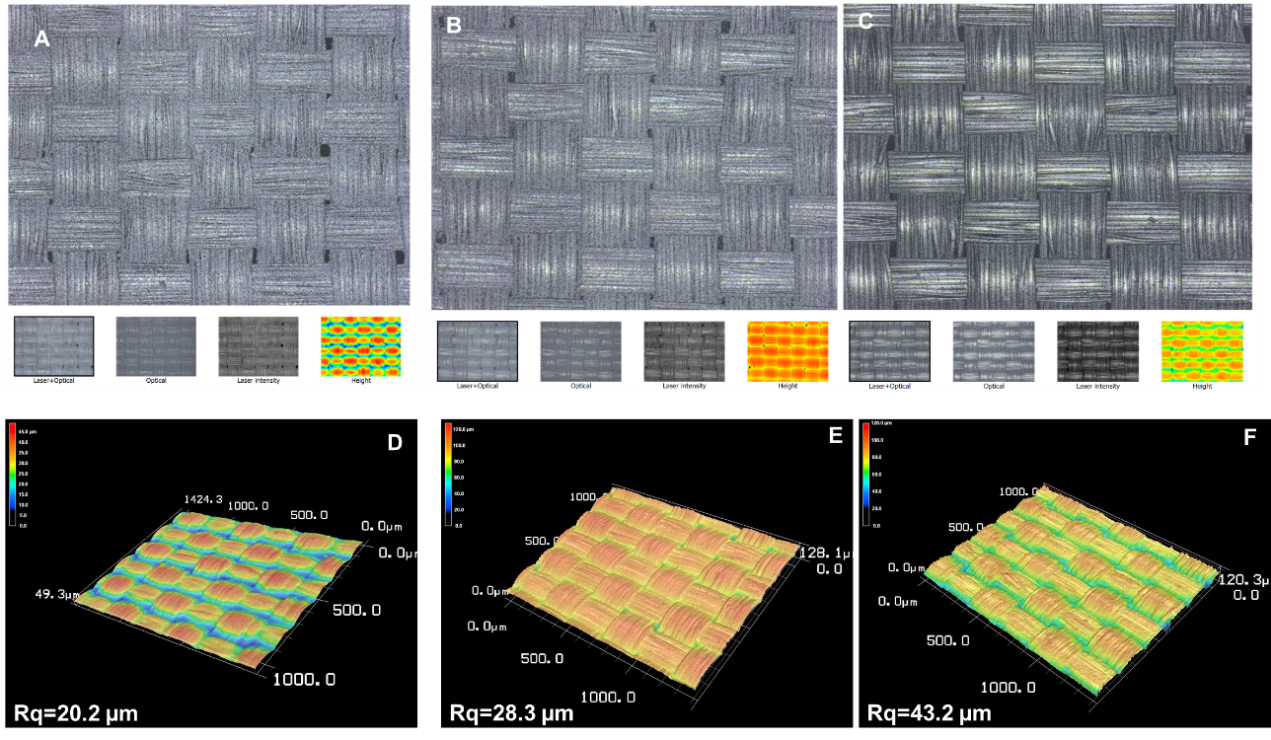


Figure. S4 . The 3D surface topography of ERL1(A&D), ERL2(B&E), and ERL3(C&F).


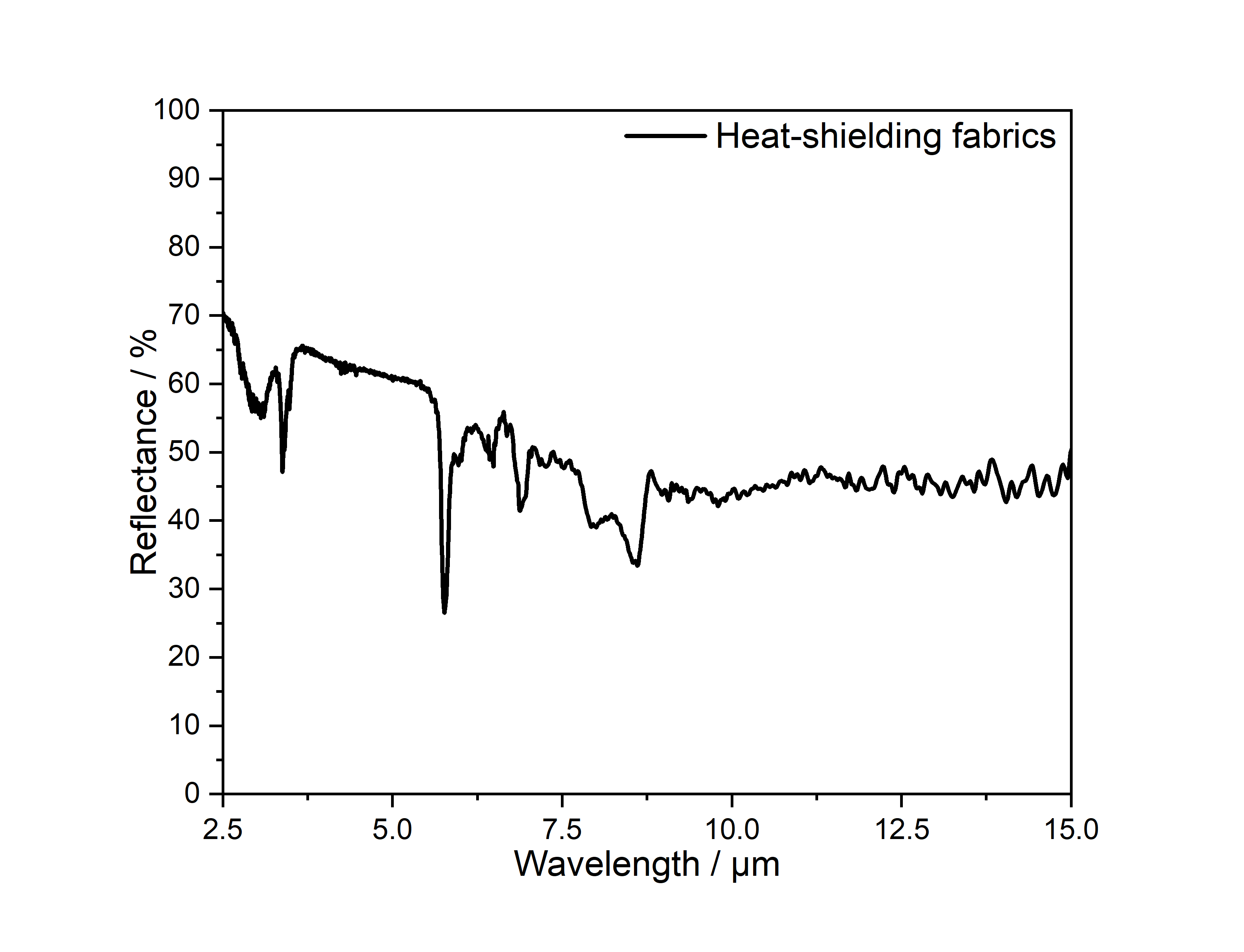


Figure. S5. The infrared reflectance of heat-shielding fabric in the range of 2.5-15 µm.


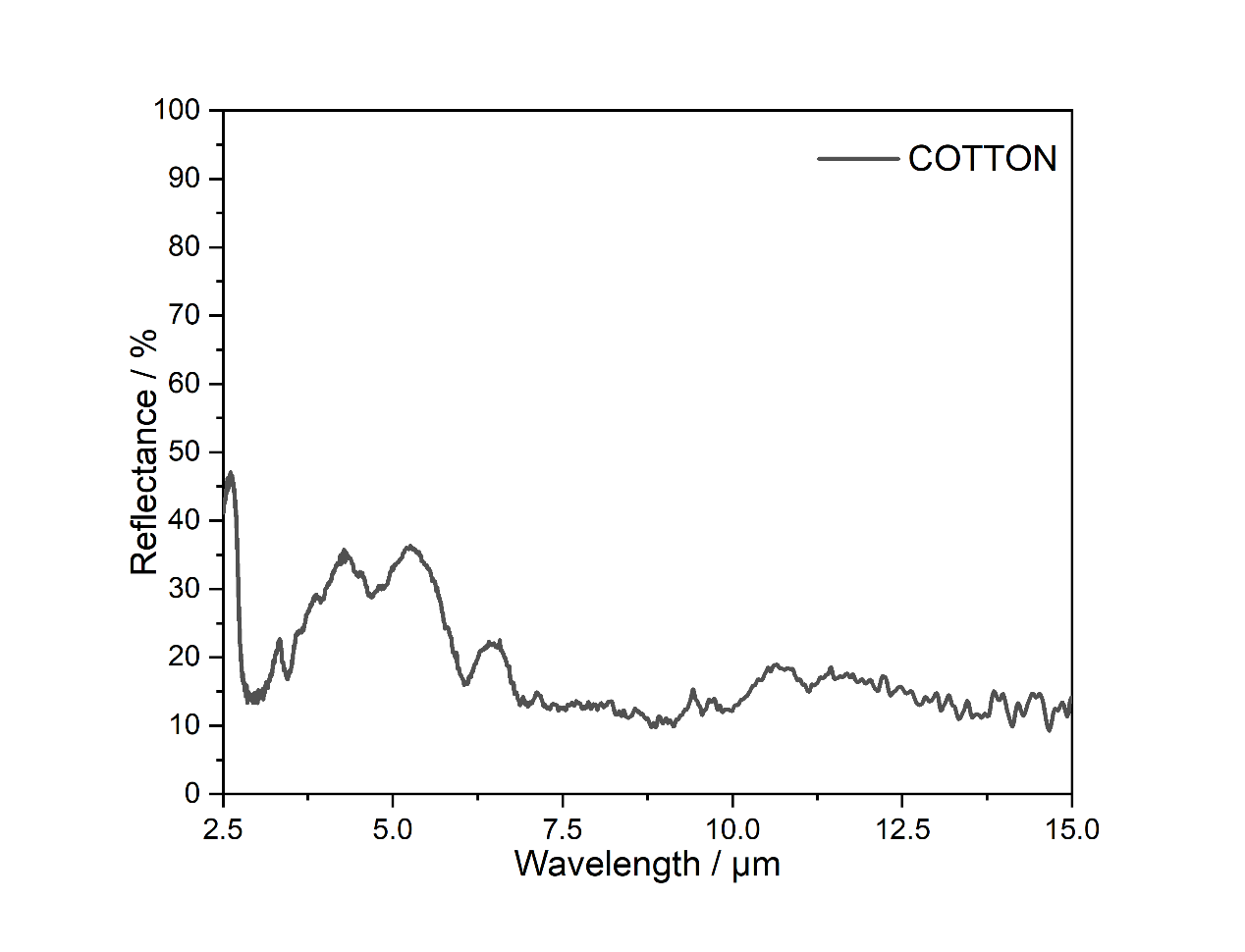


Figure. S6. The infrared reflectance of white cotton in the range of 2.5-15 µm.


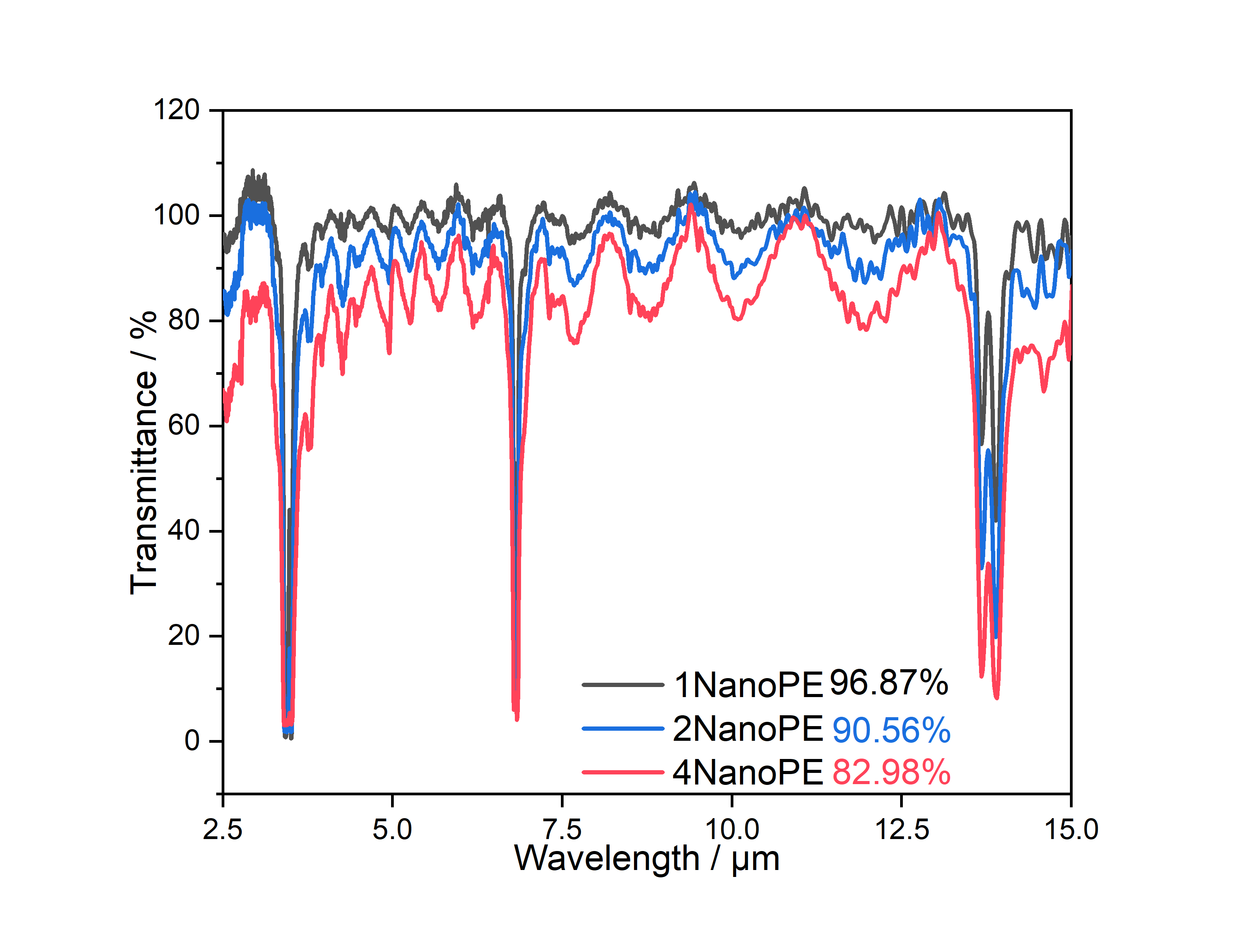


Figure. S7. Infrared transmittance of nanoPE.


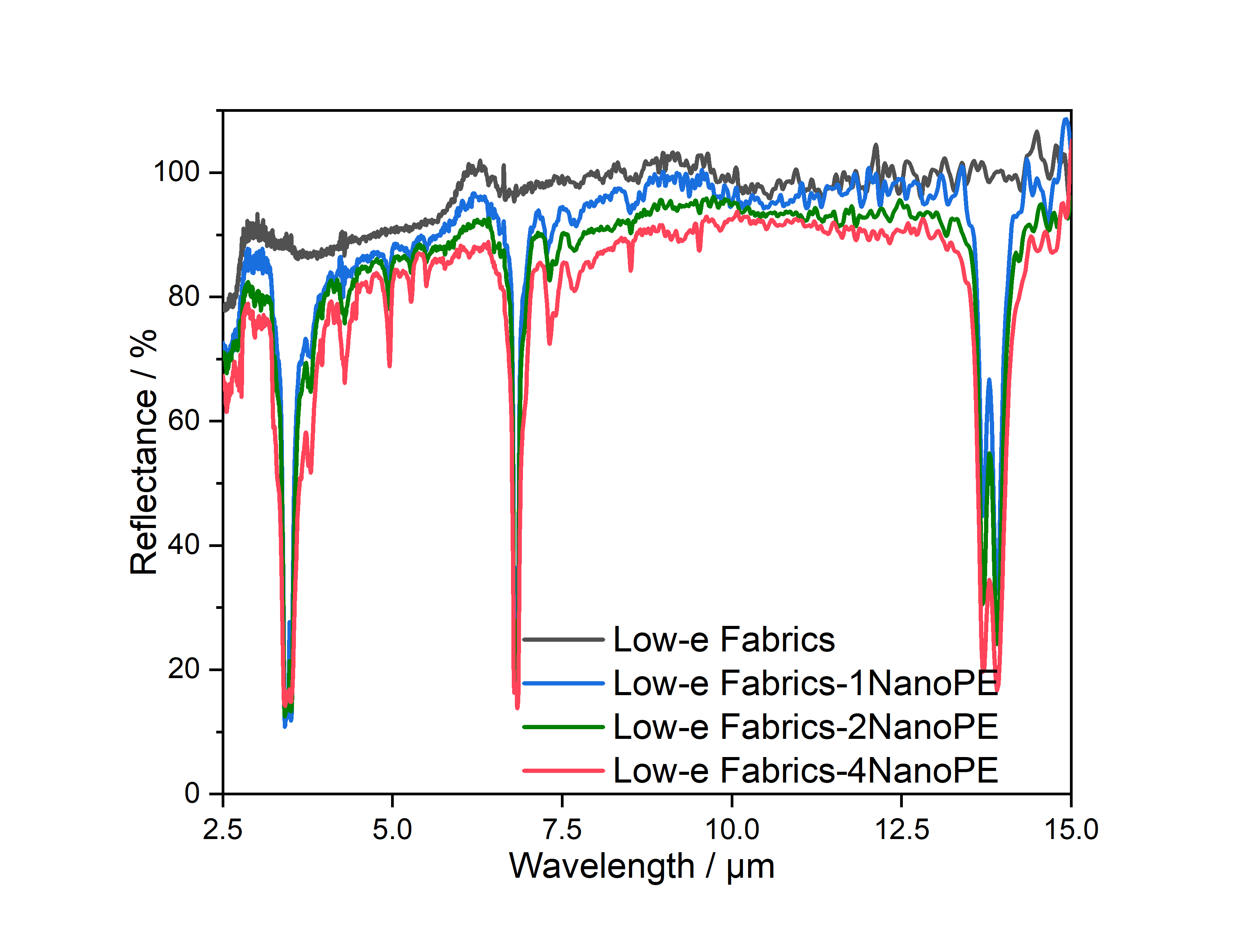


Figure. S8. Infrared reflectance of bare low-e fabric and low-e fabric covered by different layers of nanoPE.

Figure. S9. Solar reflectance of low-e fabric and nanoPE at different layers.

Figure. S10. Solar reflectance of low-e and ERLs at low and high temperatures.

Figure. S11. Emissivity of ERLs at 20^o^C and 60 ^o^C.

Figure. S12. Infrared reflectance of PET and VO_2_ coated PET at 20^o^C and 60 ^o^C.

Figure. S13. Scattering cross-section of metallic VO_2_ particles at different radius.

Figure. S14. Solar reflection and transmission of commercial white cotton.


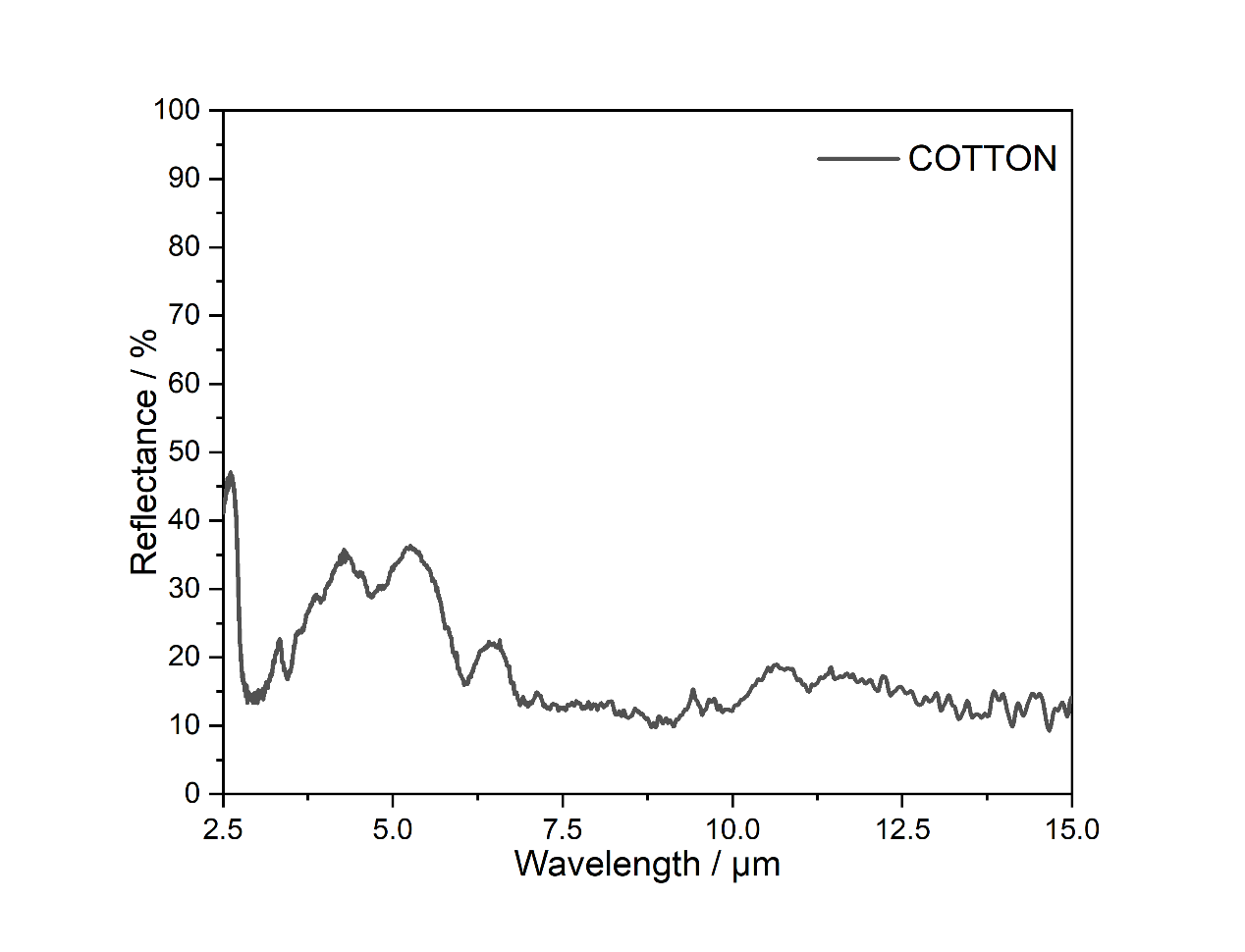


Figure. S15. The infrared reflectance of white cotton in the range of 2.5-15 µm.

Figure. S16. The infrared reflectance spectrum of ERL3 at insulating and metallic states after 0, 1000 and 2000 cycles of abrasion

| Figure S1. The ICP-OES report | | | | | | | |
| --- | --- | --- | --- | --- | --- | --- | --- |
| Element | Sampling volume m_0_/g | Constant volume V_0_ /mL | Target element concentration C_0_ /mg/L | Dilution factor f | Element concentration of digestion solution C1 mg/L | Sample element content Cx mg/kg | Sample element content W/ wt% |
| V | 0.0131 | 10 | 7.025 | 100 | 702.5 | 536259.54 | 53.63% |
| V | 0.0131 | 10 | 7.2 | 100 | 720 | 549618.32 | 54.96% |
| V | 0.0131 | 10 | 7.143 | 100 | 714.3 | 545267.18 | 54.53% |
| W | 0.0131 | 10 | 4.7681 | 10 | 47.6811 | 36359.85 | **3.64%** |
| W | 0.0131 | 10 | 4.8208 | 10 | 48.208 | 36758.7 | **3.68%** |
| W | 0.0131 | 10 | 4.7553 | 10 | 47.553 | 36329.69 | **3.63%** |

| Table S2. The XPS report | | | | | | | | |
| --- | --- | --- | --- | --- | --- | --- | --- | --- |
| Name | Start BE | Peak BE | End BE | Height CPS | FWHM eV | Area (P) CPS.eV | Area (N) TPP-2M | Atomic % |
| C1s | 297.98 | 283.9 | 279.18 | 36942.2 | 1.78 | 87746.94 | 1229.71 | 34.91 |
| O1s | 544.98 | 529.3 | 525.18 | 106603.85 | 1.6 | 233156.3 | 1349.82 | **38.32** |
| V2p | 539.98 | 529.28 | 507.18 | 109017.54 | 1.61 | 499848.67 | 898.27 | **25.5** |
| W4f | 47.98 | 41.26 | 28.18 | 9277.19 | 3.44 | 40715.92 | 44.75 | **1.27** |

Reference

1. Rasch, W.; Samson, P.; Cote, J.; Cabanac, M., **1991,** *71* (2), 590-595. DOI 10.1152/jappl.1991.71.2.590.

2. How Does Emissivity Affect Thermal Imaging? <https://www.flir.com/discover/professional-tools/how-does-emissivity-affect-thermal-imaging/> (accessed 2024-01-02).

3. Wan, C.; Zhang, Z.; Woolf, D.; Hessel, C. M.; Rensberg, J.; Hensley, J. M.; Xiao, Y.; Shahsafi, A.; Salman, J.; Richter, S.; Sun, Y.; Qazilbash, M. M.; Schmidt-Grund, R.; Ronning, C.; Ramanathan, S.; Kats, M. A., *Annalen der Physik* **2019,** *531* (10), 1900188. DOI <https://doi.org/10.1002/andp.201900188>.

4. Zhang, X.; Qiu, J.; Li, X.; Zhao, J.; Liu, L., *Appl. Opt.* **2020,** *59* (8), 2337-2344. DOI 10.1364/AO.383831.

5. Babar, S.; Weaver, J. H., *Appl. Opt.* **2015,** *54* (3), 477-481. DOI 10.1364/AO.54.000477.
